# Nighres: processing tools for high-resolution neuroimaging

**DOI:** 10.1093/gigascience/giy082

**Published:** 2018-07-04

**Authors:** Julia M Huntenburg, Christopher J Steele, Pierre-Louis Bazin

**Affiliations:** 1 Max Planck Research Group for Neuroanatomy & Connectivity, Max Planck Institute for Human Cognitive and Brain Sciences, Stephanstrasse 1a, Leipzig, 04103, Germany; 2 Neurocomputation and Neuroimaging Unit, Department of Education and Psychology, Free University of Berlin, Habelschwerdter Allee 45, Berlin, 14195, Germany; 3 Department of Neurology, Max Planck Institute for Human Cognitive and Brain Sciences, Stephanstrasse 1a, Leipzig, 04103, Germany; 4 Cerebral Imaging Center, Douglas Mental Health University Institute, 6875 LaSalle Boulevard, Montreal, Quebec, H4H 1R3, Canada; 5 Department of Psychology, Concordia University, 7141 Sherbrooke West, Montreal, Quebec, H4B IR6, Canada; 6 Department of Neurophysics, Max Planck Institute for Human Cognitive and Brain Sciences, Stephanstrasse 1a, Leipzig, 04103, Germany; 7 Psychology Department, University of Amsterdam, Nieuwe Achtergracht 129B, Amsterdam, 1018 WT, Netherlands

**Keywords:** neuroimaging in python, high-resolution MRI, ultra-high field MRI, laminar MRI, python Java integration

## Abstract

With recent improvements in human magnetic resonance imaging (MRI) at ultra-high fields, the amount of data collected per subject in a given MRI experiment has increased considerably. Standard image processing packages are often challenged by the size of these data. Dedicated methods are needed to leverage their extraordinary spatial resolution. Here, we introduce a flexible Python toolbox that implements a set of advanced techniques for high-resolution neuroimaging. With these tools, segmentation and laminar analysis of cortical MRI data can be performed at resolutions up to 500 μm in reasonable times. Comprehensive online documentation makes the toolbox easy to use and install. An extensive developer’s guide encourages contributions from other researchers that will help to accelerate progress in the promising field of high-resolution neuroimaging.

## Background

Recent advances in ultra-high field (7 Tesla [T] and above) magnetic resonance imaging (MRI) make it possible to image the entire human brain at an unprecedented level of detail [1]. Submillimeter resolutions and quantitative metrics reveal fine-grained variations in structure and function that were previously undetectable *in vivo*. This information allows researchers to ask new questions about the human brain. Examples include investigation of intracortical myelin (e.g., [2-5]), the laminar organization of the cortical sheet (e.g., [6-10]), feedforward and feedback patterns in cortical connections [11, 12], and the detailed description of small cortical and subcortical structures [13, 14] and their function [15].

While ultra-high field scanners have become increasingly available and the first open 7T MRI datasets have been released [16-18], software tools still lag behind. Standard neuroimaging software packages are often not designed to handle the growing data size and new quantitative contrasts. Three-dimensional MRI data grows as a cube of its resolution, and computational complexity generally ranges from *O*(*N*log *N*) to *O*(*N*^2^). Therefore, a change in spatial resolution from 1 mm to 0.5 mm easily entails an increase in computational requirements by a factor of 15 to 60, depending on the methods used. Moreover, new applications such as laminar analysis have only become possible with higher resolutions and are not implemented in many existing software packages.

CBS High-Res Brain Processing Tools (CBS Tools) is a software suite that addresses this gap by providing cutting-edge methods for efficient processing of MR images at submillimeter resolution [19]. For example, CBS Tools implements routine cortical segmentation at resolutions as high as 400 μm; processing of quantitative MRI sequences such as magnetization prepared two rapid acquisition gradient echoes (MP2RAGE), quantitative multi-parameter mapping (MPM), or quantitative susceptibility mapping [19]; laminar analysis [7]; and small vessel segmentation [20]. While this software has been well received as a key tool set for quantitative and high-resolution neuroimaging, its adoption has been slowed by the complex infrastructure it builds on. CBS Tools was developed in Java as a set of plug-ins for the MIPAV software package [21] and the JIST pipeline environment [22]. The MIPAV and JIST framework provides a graphical interface for building analysis pipelines and implements many convenient tools; however, it comes with a complex installation procedure, heavy dependencies, and limited documentation. More importantly, it is difficult to integrate with other popular neuroimaging tools, limiting its software ecosystem.

Meanwhile, a range of versatile, interoperable open-source packages for the analysis of neuroscientific data has been developed using the increasingly popular programming language Python [23]. For example, Nipy [24] is a community of practice devoted to the use of Python in the analysis of neuroimaging data, encompassing popular tools such as Nibabel [25], Nipype [26], Nilearn [27], and many others.

Here, we present Nighres^1^, a new toolbox that makes the quantitative and high-resolution image-processing capabilities of CBS Tools available in Python. Nighres is a user-friendly Python package that interfaces with CBS Tools while avoiding the JIST and MIPAV dependency tree. It facilitates integration with other Python-based neuroimaging tools and interactive data exploration, e.g., in Jupyter notebooks [28. Nighres features comprehensive online documentation with usage examples that are based on publicly available datasets. An extensive developer’s guide encourages external contributions. With this new package, we aim to make the functionality of CBS Tools accessible to a wider community, highlight the potential of new high-resolution image-processing methods, and foster collaboration in this emerging field.

## Implementation

### Architecture and design

The Nighres package consists of two core Python modules. The module cbstools contains the original CBS Tools Java classes that have been encapsulated using the JCC package [29]. JCC encapsulates the Java code with C++ code to make it accessible to the Python interpreter and produces a complete Python extension module. The module nighres includes the Python interfaces that are exposed to the user. It is organized in submodules that represent different application areas.^2^ For example, the submodule *laminar*contains functions related to laminar analysis of the cortical sheet. There are currently two types of Python interfaces within these submodules:

- Functions that wrap Java classes
- Functions in pure Python

#### Functions that wrap Java classes

The initial motivation to develop Nighres was to provide a user-friendly interface to the functionality of CBS Tools, leveraging the flexibility of Python. Therefore, a majority of the current functions in Nighres constitute Python wrappers that internally execute the original CBS Tools Java classes. These functions generally adhere to the following basic structure (a simple example can be found in the function *probability_to_levelset*):

- Evaluate input parameters
- Start Java virtual machine
- Initiate Java class through JCC wrapper
- Load input data and cast to Java array
- Pass additional parameters to Java class
- Execute Java class
- Collect outputs of Java class and cast back
- Return outputs (optional: save outputs)

Thus, the actual processing still relies on the same optimized Java code as in the original CBS Tools. However, since the Nighres function takes care of the interfacing between Python and Java, the user interacts only with Python code.

#### Functions in pure Python

Our long-term vision is for Nighres to become a central platform for new high-resolution image processing tools as they are developed. As discussed above, Python is rapidly becoming the most popular programming language in the neuroimaging community [23]. The modular design of Nighres allows for easy integration of pure Python processing routines, meaning that new functions can be contributed without the need to interact with Java or to learn about the JCC-based wrapping procedure. In addition, it is possible to integrate useful tools from other neuroimaging software that have been (or can be) wrapped in Python, e.g., using Nipype [26]. Currently, Nighres includes a core set of Python functions for input and output, parameter handling, and file naming to simplify function calls and minimize the integration burden for new methods.

### Data handling

Data handling within Nighres follows established and widely used standards in the neuroimaging community to ensure maximal interoperability. Where possible, Nighres uses the Nibabel package for handling imaging data [25]. Input and output functions are designed to automatically recognize and load the most commonly used data formats, while maintaining flexibility to accommodate loading of nonstandard data formats using custom scripts. Data are internally represented as Nibabel *Nifti1Images* (volumes) or Python dictionaries (surfaces) and can be passed in the form of file names or memory objects. Processing results are returned as memory objects; functions with multiple outputs return a dictionary storing the different outputs. Outputs can also be saved to disk. For saving, modifiers are appended to the output file names that refer to the name of the function and the specific output (e.g. *_layering_depth* for the continuous depth output of the layering function). Output names can be set to have a specific prefix or, by default, append modifiers to the main input file name.

### Distribution

While both Python and Java are cross-platform languages, the JCC package encapsulates CBS Tools’ Java classes with C++ code and thus makes the compilation platform specific. We implemented an automated build script that compiles the original CBS Tools Java code and builds the wrappers using JCC. We set up continuous integration using Travis CI [30] to test the build on any changes to the code base on Github and, for any tagged releases, deploy the package to the Python Package Index [31]. The user can then download the package, run the fully automated build script to recompile the Java code and C++ wrappers on their platform, and finally use the pip installer [32] to install the modules and all their dependencies. Subsequently, Nighres can simply be imported into any Python environment. We also provide a container allowing users to test Nighres in a preset environment, without actually installing it on their system. For this option, the user only has to install Docker [33], a lightweight container platform that runs on Linux, Windows, and Mac OS X. The Nighres Dockerfile [34] can then be used to build an Ubuntu 14 Trusty Docker image that contains a suitable Java installation, Nighres, and Jupyter Notebook.

#### Dependencies

One goal of Nighres was to reduce external dependencies. We therefore restricted the required packages for Nighres’ core functionality to Nibabel for reading and writing of common neuroimaging data formats [25], and Numpy for efficient manipulation of data arrays [35]. The functions wrapping CBS Tools code require the CBS Tools Java library as well the Java matrix manipulation [36] and Apache Commons Math [37] libraries. However, these libraries are automatically recompiled, wrapped, and installed from the CBS Tools github repository [38] upon installation of Nighres. Our example workflows use Nilearn’s [27] plotting functionality for visualizing their results but will automatically skip plotting if Nilearn is not installed.

#### Support files

Nighres automatically installs all essential support files including statistical atlases for brain segmentation, look-up tables for topological constraints, templates for high-resolution spatial normalization, and a cerebellar lobular atlas [39]. Example data from publicly released 7T datasets are hosted on the Nighres project page [40] at the neuroimaging informatics tools and resources clearinghouse (NITRC, [41]). The data are automatically downloaded when running the example workflows.

### Documentation

Beyond functional code, clear and concise documentation is one of the most important drivers of software use and longevity. Nighres’ online documentation [42] was implemented using the Sphinx documentation tool [43]. The online content is automatically generated from the original function docstrings, which are written according to the Numpy/Scipy documentation guidelines [44]. This design ensures that the documentation stays up-to-date with minimal overhead for developers and is intuitive for users. Extensive example workflows provide users with easily understandable and reproducible code (see section *Usage example* below). Finally, the online documentation contains an in-depth developer’s guide that leads contributors through all steps necessary to submit code changes, new Python functions, or CBS Tools wrappers to the Nighres github repository. We aimed to write a guide that makes it feasible for any researcher working with high-resolution neuroimaging data to contribute to Nighres, even without much previous experience in software development.

### Functionality

Nighres contains a set of advanced functions that are not commonly implemented in neuroimaging software and/or have been optimized toward the specific demands of processing high-resolution and quantitative neuroimaging data. In this section, we provide an overview of the major features that are currently implemented. Their application will be demonstrated in the subsequent section, which also indicates example computation times. A more in-depth discussion of the individual algorithms and their performance can be found in the original references listed for each function.

#### MP2RAGE skull-stripping

This fast skull-stripping algorithm has been optimized for quantitative images acquired at 7T using the MP2RAGE sequence [45]. See [19] for details.

#### Multiple object geometric deformable model segmentation

Multiple object geometric deformable model segmentation (MGDM) is a whole-brain tissue classification method designed to routinely process datasets at resolutions up to 400 μm. A variety of inputs (MP2RAGE at 3T, 7T, and 9.4T; MPM at 3T and 7T; T1-, T2-, and diffusion-weighted images) as well as multiple inputs are accepted. This atlas-guided method uniquely preserves the topological properties and relationships of all 25 classified brain structures. See [19, 46, 47] for details.

#### Cortical reconstruction using implicit surface evolution

Cortical reconstruction using implicit surface evolution (CRUISE) provides a precise and efficient method to extract cortical surfaces from high-resolution volumetric data based on level set representations (see next subsection). A distinguishing feature of this algorithm is the careful modeling of sulcal fundi. CRUISE can be applied to cerebral and cerebellar cortices and to data with partial brain coverage. See [48] for details.

#### Level set creation

This function creates level sets from probabilistic or deterministic tissue classifications. Level sets are signed distance functions that can be used for representing cortical surfaces in voxel space instead of triangular meshes. Such representations have favorable mathematical properties, avoid mesh sampling problems, and facilitate the integration of volumetric and surface data. Several Nighres functions rely on level sets internally. See [19] for details.

#### Equivolumetric layering

Nighres implements an equivolumetric technique for modeling intracortical laminae. This approach accounts for the dependency of layer thickness on local curvature by preserving the volume of cortical segments (cf. [49]). The resulting cortical depth estimates represent an intracortical coordinate system that is anatomically more accurate than commonly applied equidistant or Laplacian approaches. Intracortical surfaces are represented as level sets. See [50] for details.

In addition to the aforementioned functions, we are currently preparing to migrate CBS Tools’ multimodal surface registration algorithm [51] and nonlinear deformation utilities, as well as algorithms for topology correction [52] and vascular segmentation [20], into the Nighres package.

## Usage Example

Here, we present a Nighres usage example. It shows how to obtain a tissue classification and cortical depth estimation from MP2RAGE data, acquired at 7T with a resolution of 0.7 mm isotropic. The pipeline contains the following steps:

1. Downloading the open MP2RAGE data set from NITRC
2. Removing the skull and creating a brain mask
3. Atlas-guided tissue classification using MGDM [46]
4. Extracting the cortex of one hemisphere
5. Cortical reconstruction using CRUISE [48]
6. Equivolumetric modeling of intracortical laminae [50]

**Figure 1:**
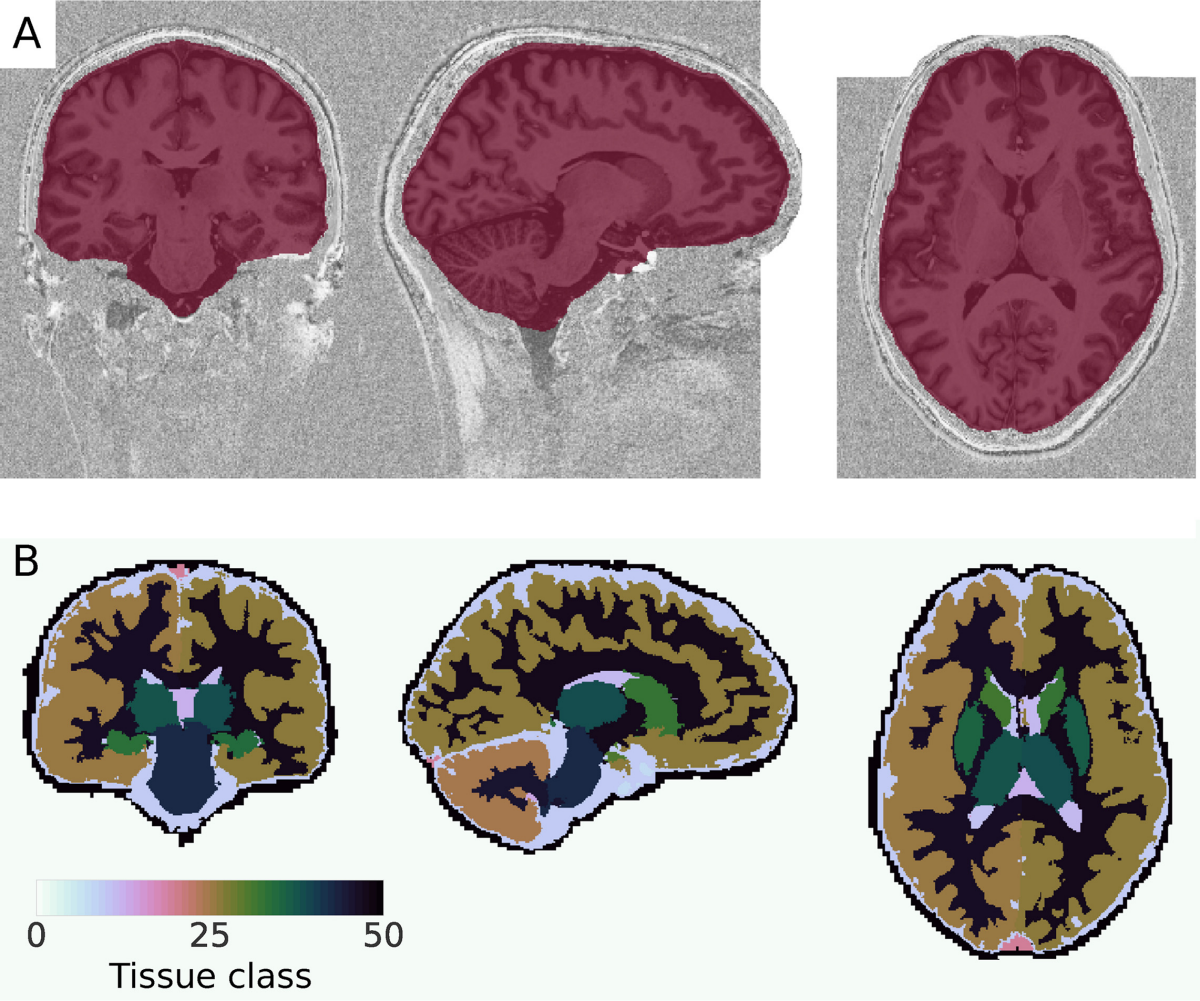
Tissue classification from MP2RAGE data. **(A)** The brain mask obtained from skull stripping. Note that the white rectangles in the image occur because the data has been ”defaced” for anonymization. **(B)** The result of the MGDM tissue classification. Visualized using Nilearn [27].

**Figure 2:**
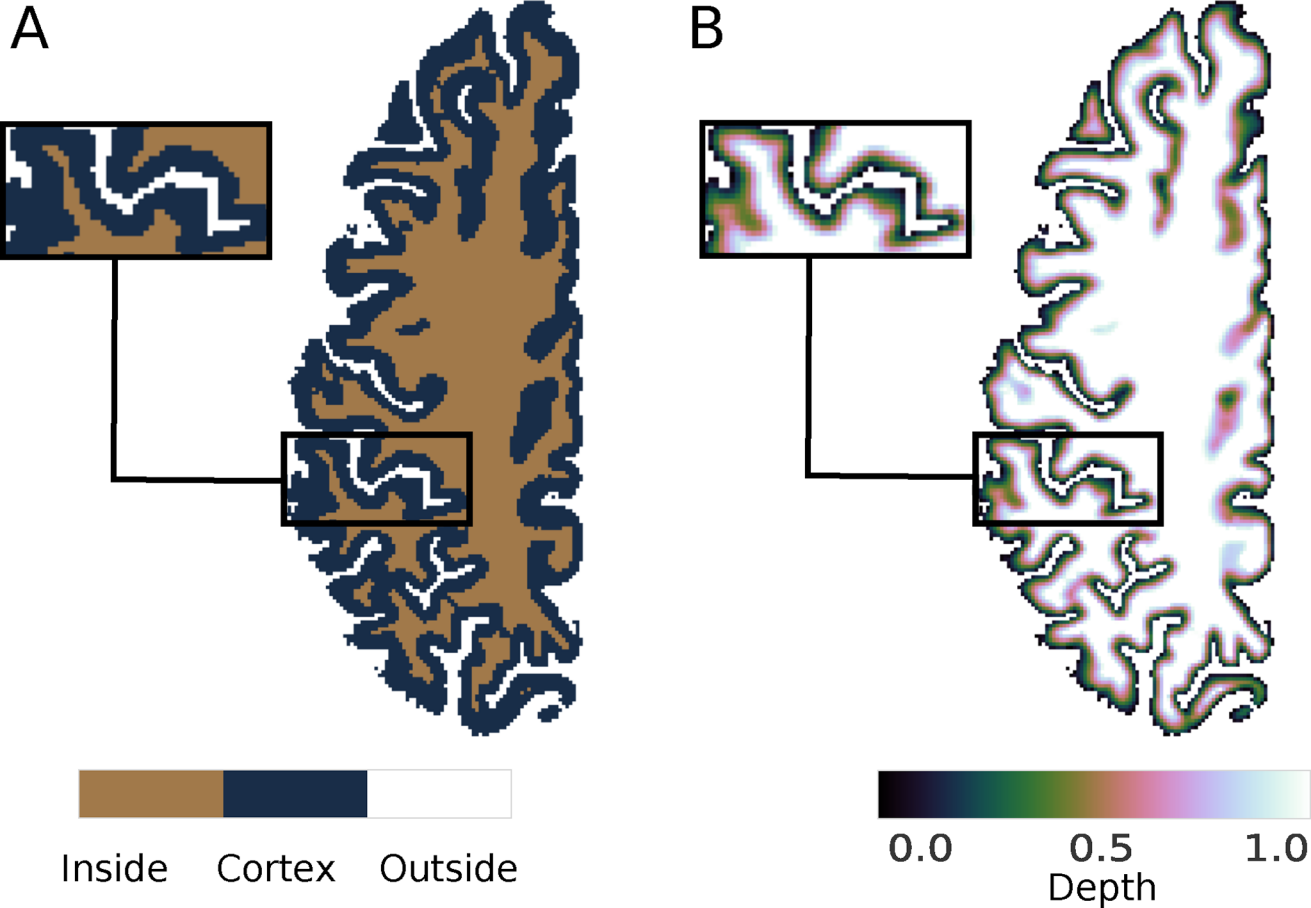
Cortical surface reconstruction and depth estimation. **(A)** Topology-constrained reconstruction of the boundaries between the cortical gray matter (cortex, blue), the cerebrospinal fluid (outside, white), and the white matter (inside, brown) using CRUISE [48]. (**B**) Intracortical depth estimated using an equivolumetric approach [50]. Visualized using Nilearn [27].

The outputs of the plotting functions are shown in Figs. 1 and 2. Average computation times for the different processing steps in this example are indicated in Table 1. They were determined on a standard laptop (8 GB random access memory, i7-5500U dual core processor, 4 MB cache, 3 GHz maximum frequency) using Python’s timeit module [53].

**Table 1:** Computation times for usage example

Processing step	Duration
Skull stripping	1 minute 8 seconds (8 seconds)
MGDM tissue classification	6 minutes 58 seconds (30 seconds)
CRUISE surface reconstruction	1 minute 57 seconds (3 seconds)
Equivolumetric layering	1 minute 23 seconds (6 seconds)

Shown are average durations over 10 repetitions (with standard deviations in brackets), determined on a standard laptop. See main text for details.

### Import and download

First, we import nighres and the os module to set the output directory

import nighres, os

out_dir = os.path.join

(os.getcwd(),“nighres_examples/tissue_classification”)

We also import Nilearn’s plotting functions. If Nilearn is not installed, plotting will be skipped in the online examples.

from nilearn import plotting

Now, we download an example MP2RAGE dataset that is hosted on NITRC. It is the structural scan of the first subject, first session of the 7T test-retest dataset published in [17].

dataset = nighres.data.download_7T_TRT(out_dir)

### Skull stripping

The first processing step is skull stripping. The brain mask is calculated based on the second inversion image of the MP2RAGE sequence. For convenience, we can also input the quantitative T1 map and the T1-weighted image, to which the calculated brain mask will then be applied. We save the outputs in the out_dir specified above and use a subject ID as the base file name.

skullstrip_results = nighres.brain.mp2rage_skullstripping

(second_inversion=dataset[“inv2”],

t1_weighted=dataset[“t1w”],

t1_map=dataset[“t1map”],\thinspace

save_data=True, output_dir=out_dir

file_name=“sub001_sess1”)

To check if the skull stripping worked well, we plot the brain mask on top of the original image (Fig. 1A). Nighres, like Nilearn, uses Nibabel’s *Nifti1Image* object to pass data internally. Therefore, we can directly pass the outputs to Nilearn’s plotting functions without saving and reloading. Alternatively, the images stored in out_dir can be opened in any common interactive viewer that can read the Nifti data format.

plotting.plot_roi(skullstrip_results[“brain_mask”],

dataset[“t1w”], cut_coords=[15, 25, 30],

annotate=False, black_bg=False, draw_cross=False,

cmap=“PuRd_r”)

(We hereafter omit the plotting code; it can be found in the online documentation.)

### MGDM tissue classification

Next, we use the masked data as input for tissue classification with the MGDM algorithm [46]. MGDM works with a single contrast but can be improved with additional contrasts. In this case, we use the T1-weighted image as well as the quantitative T1 map.

mgdm_results = nighres.brain.mgdm_segmentation

(contrast_image1=skullstrip_results[“t1w_masked”],

contrast_type1=“Mp2rage7T”,

contrast_image2=skullstrip_results[“t1map_masked”],

contrast_type2=“T1map7T”,

save_data=True, output_dir=out_dir,

file_name=“sub001_sess1”)

The topology-constrained segmentation that MGDM creates is shown in Fig. 1B.

### Cortical surface reconstruction

First, we extract the regions needed for cortical reconstruction from the MGDM output. The outputs are membership functions for the following regions: the gray matter cortex (“region"), the underlying white matter (with filled subcortex and ventricles; “inside"), and the surrounding cerebrospinal fluid (with masked regions; “background").

cortex = nighres.brain.extract_brain_region

(segmentation=mgdm_results[“segmentation”],

levelset_boundary=mgdm_results[“distance”],

maximum_membership=mgdm_results[“memberships”],

maximum_label=mgdm_results[“labels”],

extracted_region=“left_cerebrum”,

save_data=True, output_dir=out_dir,

file_name=“sub001_sess1_left_cerebrum”)

Next, we use the extracted data as input for cortical reconstruction with the CRUISE algorithm [48]. CRUISE uses the membership functions as a guide and the white matter mask as a (topologically spherical) starting point to grow refined boundaries between the gray and white matter and the gray matter and the cerebrospinal fluid.

cruise = nighres.cortex.cruise_cortex_extraction

(init_image=cortex[“inside_mask”],

wm_image=cortex[“inside_proba”],

gm_image=cortex[“region_proba”],

csf_image=cortex[“background_proba”],

normalize_probabilities=True,

save_data=True, output_dir=out_dir

file_name=“sub001_sess1_left_cerebrum”)

The topology-constrained segmentation with refined boundaries that CRUISE created is shown in Fig. 2A.

### Modeling of intracortical laminae

Finally, we use the gray–white matter boundary (GWB) and cerebrospinal fluid–gray matter boundary (CGB) from CRUISE to compute cortical depth and model intracortical laminae. Importantly, the equivolumetric approach implemented in Nighres accounts for the dependency of layer thickness on cortical folding (for an in-depth discussion, see [50]).

depth = nighres.laminar.volumetric_layering

(inner_levelset=cruise[“gwb”],

outer_levelset=cruise[“cgb”],

n_layers=4,

save_data=True, output_dir=out_dir,

file_name=“sub001_sess1_left_cerebrum”)

Fig. 2B shows the continuous equivolumetric depth estimate. The function call also outputs discrete representations of the modeled laminae as well as level sets describing the intracortical surfaces.

In summary, this example implements a complete workflow for advanced processing of a quantitative MR contrast at high spatial resolution (voxel size = 0.7 mm isotropic). With the openly available and automatically downloaded data, any user can try out Nighres’ functionality immediately after installation and then adapt the code for their own use case. The examples can be found in our online documentation [54], where the code can be downloaded as Python scripts or Jupyter notebooks.

## Discussion

The availability of high-resolution and quantitative MRI data and the interest in new research directions that these data enable are rapidly growing (e.g., [55, 56]). At the same time, image processing tools required to leverage the new level of spatial detail provided by this data are scarce. We developed a Python toolbox that specializes in processing high-resolution brain imaging data. It has been designed with two key purposes in mind:

- to provide the neuroimaging community with user-friendly access to cutting-edge high-resolution image processing tools
- to create a flexible framework that can be extended by other researchers, along with thorough instructions on how to contribute

### Comparison to other tools

Most major neuroimaging packages are optimized for data with a maximum spatial resolution of 1 mm isotropic. Only recently have some extensions and new tools for processing of high-resolution data begun to emerge, which will be discussed in the following section [57].

#### Freesurfer

Freesurfer is a popular open-source package for analyzing cortical surface data [58, 59]. It is robust, well documented, and applicable across platforms. By default, Freesurfer resamples the input data to a spatial resolution of 1 mm isotropic, obliterating the advantages of higher-resolution data. The latest Freesurfer release includes an option for processing at submillimeter resolution [60]. However, this option is still under development and tested only for a resolution of 0.75 mm [61]. A Matlab routine for laminar analysis of high-resolution MRI data using Freesurfer has been proposed as well [62]. Here, intracortical surfaces are evolved as triangular meshes starting from the gray-white matter boundary in an equidistant fashion. This approach can cause errors and mesh irregularities, especially closer to the pial surface, and does not take into account the known dependency of layer thickness on local curvature.

More generally, Freesurfer’s robustness and ease of use come at the cost of strict requirements for data organization (e.g., imposed directory structure, native file format) and limited flexibility in the adaptation of individual processing steps for new applications. While it provides excellent pipelines for standard processing of T1- or T2-weighted whole brain scans, it is not optimized for processing nonstandard data such as quantitative T1 maps or images with partial brain coverage. At the same time, replacing individual processing steps with customized algorithms, combining Freesurfer with other tools, or applying manual corrections can be challenging even for experienced users. Therefore, while Freesurfer will likely play an important role, as ultra-high field imaging becomes more abundant, it currently lacks the flexibility required for the active and collaborative development of new techniques in this emerging field.

#### BrainVoyager

Another software that has recently extended its functionality to the specific demands of high-resolution image processing is BrainVoyager [63]. A new pipeline, comprehensively described in a recent publication [64], enables laminar and columnar analyses of 9.4T data. Intracortical surfaces are modeled following the equivolumetric approach through the evolution of regular grids for small regions of interest. Unfortunately, BrainVoyager is a commercial software with closed source code. In addition to the financial aspect of buying a license, this also entails that details of the applied algorithms are not transparent and the software cannot be adapted by users.

#### LAYNII

LAYNII is a set of highly optimized C++ tools for laminar analysis of high-resolution fMRI data with partial brain coverage [12,65]. Equivolumetric layering is available for slices without 3D curvature. The implementation in C++ enables fast processing but has the disadvantage that fewer researchers can adapt or contribute code, as compared to high-level languages such as Python. LAYNII also lacks documentation, making it hard for new users to adopt it.

LAYNII is a good example of an advanced toolbox that serves a specific purpose and could benefit from being combined with a more comprehensive and well-documented software framework for high-resolution image processing. It will be crucial in the future to synchronize Nighres with more specialized projects such as LAYNII and make their integration as easy as possible.

#### CBS Tools

Nighres evolved out of CBS Tools, a suite of Java tools providing dedicated open-source methods for high-resolution and quantitative image processing [19]. This includes specialized techniques such as equivolumetric layering [50] and multimodal surface registration [51], as well as versions of more common applications such as tissue classification that have been optimized for high-resolution data and quantitative contrasts. While many standard processing algorithms in neuroimaging grow at a log-linear (*O*(*N*log *N*)) or even quadratic (*O*(*N*^2^)) rate with data size, CBS Tools’ algorithms approach linear rates (*O*(*N*)) or use noniterative solutions (for details, see [19]). CBS Tools can thus routinely operate on data at resolutions of up to 0.5 mm isotropic.

As described in the introduction, CBS Tools’ complex design and heavy dependencies make installation and handling challenging and impede contributions from other researchers. For a previous project, we presented simple Python wrappers for selected CBS Tools functions [66]. Here, we described a comprehensive software framework that has evolved out of these initial attempts. With Nighres, we present a flexible and user-friendly implementation of CBS Tools’ functionality, which eliminates the dependency on MIPAV and JIST. This approach provides a significant improvement in usability while preserving the excellent performance of CBS Tools. Another major advance of Nighres compared to CBS Tools is its extensive online documentation. In addition to explaining every function’s inputs and outputs, it provides carefully documented usage examples with step-by-step instructions of how the different tools can be combined to create complete processing pipelines. The implementation in Python along with a detailed developer’s guide facilitate adaptation and extension of the existing tools by other researchers.

We gave an example of Nighres’ performance in the previous section (see Table 1). To put this example into perspective, consider Freesurfer’s recon-all command, probably the most common approach for whole brain tissue classification and cortical surface reconstruction. This command processes a whole brain image at 1 mm isotropic resolution within a few hours. In comparison, the Nighres pipeline presented above achieves tissue classification and segmentation plus cortical layering at 0.7 mm isotropic resolution (roughly corresponding to a 3-fold increase in data size compared to 1 mm isotropic) in less than 15 minutes.

### Future directions

The current implementation of Nighres contains a set of cutting-edge methods; however, rapid methodological advances are to be expected in the dynamic field of high-resolution neuroimaging. We therefore designed Nighres as a transparent software platform through which newly developed methods can be made available to the community and improved collaboratively. New or existing tools can easily be added in a variety of formats, depending on the specific requirements of the operation and the preferences of the developer. The extensive developer’s guide aims to encourage contributions, even from researchers without extensive experience in software development.

We intend to closely integrate our package with the existing community around neuroimaging tools in Python. To this end, we adopted standardized objects for internal data handling, which can easily be exchanged with other tools. An example is the seamless visualization of Nighres outputs using Nilearn’s [27] plotting functions, as showcased in the usage example (Figs. 1 and 2).

A major limitation of the current package is that it has been developed and tested for common Linux platforms only. The C++ code generated by JCC to interface with CBS Tools’ Java classes makes the compilation platform dependent. We addressed this issue by providing an automated build script that recompiles the code upon installation. While this process has only been tested on Linux, the design makes future adaptation to Mac OS X platforms straightforward. Support for Windows is not currently planned. However, the provided Dockerfile enables usage of Nighres in a container on any platform that supports Docker.

Many future extensions of the current package can be envisioned. In addition to integrating more of the original CBS Tools functions, a main goal is to extend functionality with new tools coded directly in Python and potentially to replace the Java dependency altogether. To ensure efficient processing of the large amount of data, this might require increasing Python’s performance, e.g., using Numba [67]. Another goal is to provide integration with tools for parallel processing and job management on compute clusters.

### Conclusion

We developed a user-friendly and well-documented Python package that makes cutting-edge high-resolution image processing tools available to the research community. The toolbox is easy to install and provides a comprehensive set of advanced techniques. While the current functionality is largely based on CBS Tools, we hope that the flexible framework encourages contribution of new tools, stimulates collaboration, and accelerates progress in the promising field of high-resolution neuroimaging.

## Availability and requirements

- Project name: Nighres
- Project home page: https://github.com/nighres/nighres
- Operating system(s): Linux
- Programming language: Python, Java
- Other requirements: Java≥1.7, Python≥2.7, Numpy≥1.13, Nibabel≥2.1.0
- License: Apache License 2.0
- RRID:SCR_016287

## Availability of supporting data

The datasets that support the results of this article are available in the NITRC image repository [41] under https://www.nitrc.org/frs/?group_id=1205. Snapshots of the data and code are also available in the *GigaScience* GigaDB repository [68].

## Abbreviations

CGB: cerebrospinal fluid-gray matter boundary; CRUISE: cortical reconstruction using implicit surface evolution; GWB: gray-white matter boundary; MGDM: multiple object geometric deformable model; MPM: quantitative multi-parameter mapping; MP2RAGE: magnetization prepared two rapid acquisition gradient echoes; MRI: magnetic resonance imaging; Nighres: NeuroImaginG at High RESolution; NITRC: the neuroimaging informatics tools and resources clearinghouse; T: Tesla.

## Competing interests

The authors declare that they have no competing interests.

## Funding

J.M.H. was partially funded by a stipend from Google via the Google Summer of Code 2017 Program, with the International Neuroinformatics Coordinating Facility (INCF) as the mentoring organization.

## Author Contributions

J.M.H., C.J.S., and P.L.B. contributed equally to the conceptualization of the project and writing of the manuscript. J.M.H. led and C.J.S. and P.L.B. supported software development. All authors read and approved the final manuscript.

## Supplementary Material

GIGA-D-17-00325_Original_Submission.pdf

GIGA-D-17-00325_Revision_1.pdf

Response_to_Reviewer_Comments_Original_Submission.pdf

Reviewer_1_Report_(Original_Submission) -- Laurentius Huber 1/8/2018 Reviewed

Reviewer_2_Report_(Original_Submission) -- Christin Y. Sander, Ph.D. 2/15/2018 Reviewed

## Footnotes

1
**N**euro**I**magin**G** at **H**igh **RES**olution

2 For consistency the submodule names are based on the original module organization in CBS Tools
