# Supplementary material for: Nighres: processing tools for high-resolution neuroimaging
Source: Gigascience. 2018 Jul 4;7(7):giy082. doi: 10.1093/gigascience/giy082 (PMC6065481; doi:10.1093/gigascience/giy082)
Supplement: GIGA-D-17-00325_Original_Submission.pdf [file giy082_giga-d-17-00325_original_submission.pdf]

GigaScience

Nighres: Processing tools for high-resolution neuroimaging

--Manuscript Draft--

**Manuscript Number:**	GIGA-D-17-00325
**Full Title:**	Nighres: Processing tools for high-resolution neuroimaging
**Article Type:**	Technical Note
**Funding Information:**	
**Abstract:**	With recent improvements in magnetic resonance imaging (MRI) at ultra-high fields, the amount of data collected per subject in a given MRI experiment has increased considerably. Standard image processing packages are often challenged by the size of these data and dedicated methods are needed to leverage their extraordinary spatial resolution. Here we introduce a flexible Python toolbox which implements a set of advanced techniques for high-resolution neuroimaging. With these tools, segmentation and laminar analysis of cortical MRI data can be performed at resolutions up to 500 μm in reasonable times. Comprehensive online documentation makes the toolbox easy to use and install. An extensive developer's guide encourages contributions of other researchers that will help to accelerate progress in the promising field of high-resolution neuroimaging.
**Corresponding Author:**	Julia M Huntenburg<br>Max-Planck-Institut fur Kognitions- und Neurowissenschaften<br>Leipzig, GERMANY
**Corresponding Author Secondary Information:**	
**Corresponding Author's Institution:**	Max-Planck-Institut fur Kognitions- und Neurowissenschaften
**Corresponding Author's Secondary Institution:**	
**First Author:**	Julia M Huntenburg
**First Author Secondary Information:**	
**Order of Authors:**	Julia M Huntenburg
	Christopher J Steele
	Pierre-Louis Bazin
**Order of Authors Secondary Information:**	
**Opposed Reviewers:**	
**Additional Information:**	
**Question**	**Response**
Are you submitting this manuscript to a special series or article collection?	No
**Experimental design and statistics**<br><br>Full details of the experimental design and statistical methods used should be given in the Methods section, as detailed in our Minimum Standards Reporting Checklist. Information essential to interpreting the data presented should be made available in the figure legends.<br><br>Have you included all the information	No

Powered by Editorial Manager® and ProduXion Manager® from Aries Systems Corporation

requested in your manuscript?	
If not, please give reasons for any omissions below.<br><br>as follow-up to "**Experimental design and statistics**<br><br>Full details of the experimental design and statistical methods used should be given in the Methods section, as detailed in our Minimum Standards Reporting Checklist. Information essential to interpreting the data presented should be made available in the figure legends.<br><br>Have you included all the information requested in your manuscript?<br><br>"	Not applicable as this manuscript describes software
**Resources**<br><br>A description of all resources used, including antibodies, cell lines, animals and software tools, with enough information to allow them to be uniquely identified, should be included in the Methods section. Authors are strongly encouraged to cite Research Resource Identifiers (RRIDs) for antibodies, model organisms and tools, where possible.<br><br>Have you included the information requested as detailed in our Minimum Standards Reporting Checklist?	Yes
**Availability of data and materials**<br><br>All datasets and code on which the conclusions of the paper rely must be either included in your submission or deposited in publicly available repositories (where available and ethically appropriate), referencing such data using a unique identifier in the references and in the "Availability of Data and Materials" section of your manuscript.<br><br>Have you have met the above requirement as detailed in our Minimum Standards Reporting Checklist?	Yes

Main Latex file Click here to download Manuscript main.tex

GigaScience, 2017, 1–7

doi: xx.xxxx/xxxx
Manuscript in Preparation
Technical Note

TECHNICAL NOTE

Nighres: Processing tools for high-resolution neuroimaging

Julia M Huntenburg[1,2,*], Christopher J Steele[3,4,†] and Pierre-Louis Bazin[3,5,6,7,†]

[1]Max Planck Research Group for Neuroanatomy & Connectivity, Max Planck Institute for Human Cognitive and Brain Sciences, Leipzig, Germany and [2]Neurocomputation and Neuroimaging Unit, Department of Education and Psychology, Free University of Berlin, Berlin, Germany and [3]Department of Neurology, Max Planck Institute for Human Cognitive and Brain Sciences, Germany and [4]Cerebral Imaging Center, Douglas Mental Health University Institute, Montreal, QC, Canada and [5]Department of Neurophysics, Max Planck Institute for Human Cognitive and Brain Sciences, Leipzig, Germany and [6]Social Brain Lab, Netherlands Institute for Neuroscience, Amsterdam, Netherlands and [7]Spinoza Centre for Neuroimaging, Amsterdam, Netherlands

[*]Correspondance: huntenburg@cbs.mpg.de
[†]Contributed equally

Abstract

With recent improvements in magnetic resonance imaging (MRI) at ultra-high fields, the amount of data collected per subject in a given MRI experiment has increased considerably. Standard image processing packages are often challenged by the size of these data and dedicated methods are needed to leverage their extraordinary spatial resolution. Here we introduce a flexible Python toolbox which implements a set of advanced techniques for high-resolution neuroimaging. With these tools, segmentation and laminar analysis of cortical MRI data can be performed at resolutions up to 500 µm in reasonable times. Comprehensive online documentation makes the toolbox easy to use and install. An extensive developer's guide encourages contributions of other researchers that will help to accelerate progress in the promising field of high-resolution neuroimaging.

Key words: Neuroimaging in Python; High-resolution MRI; Ultra-high field MRI; Laminar MRI; Python Java integration

Background

Advances in ultra-high field (7 Tesla and above) MRI now make it possible to image the whole brain at an unprecedented level of detail [1]. Submillimeter resolutions and quantitative metrics reveal fine-grained variations in structure and function that were previously undetectable in vivo, and allow researchers to ask new questions about the human brain. Examples include the investigation of intracortical myelin [e.g 2, 3, 4, 5], the laminar organization of the cortical sheet [e.g. 6, 7, 8, 9, 10], feedforward and feedback patterns in cortical connections [11, 12, 13] and the detailed description of small cortical and subcortical structures [14, 15] and their function [16].

While ultra-high field scanners have become increasingly available and the first open 7 Tesla MRI data sets have been released [17, 18, 19], software tools still lag behind. Standard neuroimaging software packages are often not designed to handle the growing data size and new quantitative contrasts. Three-dimensional MRI data grows as a cube of its resolution, and computational complexity generally ranges between

Compiled on: November 29, 2017.
Draft manuscript prepared by the author.

Key Points

- A toolbox dedicated to the processing of high-resolution MRI data
- Lightweight and flexible code written in Python for ease of use, expansion and integration with other tools
- Extensive documentation with developer's guide and usage examples based on open data

$O(N \log N)$ and $O(N^2)$. Therefore, a change in spatial resolution from 1 mm to 0.5 mm easily entails an increase in computational requirements by a factor of 15 to 60, depending on the methods used. Moreover, many new applications, such as laminar analysis, have only become possible with higher resolutions and are not implemented in existing software packages.

CBS High-Res Brain Processing Tools (CBS Tools) is a software suite which addresses this gap by providing cutting-edge methods for efficient processing of MR images at submillimeter resolution [20]. For example, CBS Tools implements routine cortical segmentation at resolutions as high as 400 μm, processing of quantitative MRI sequences such as MP2RAGE, MPM or QSM [20], laminar analysis [7], and small vessel segmentation [21]. While this software has been well-received as a key tool set for quantitative and high-resolution neuroimaging, its adoption has been slowed by the complex infrastructure it builds on. CBS Tools have been developed in Java as a set of plugins for the MIPAV software package [22] and the JIST pipeline environment [23]. The MIPAV / JIST framework provides a graphical interface for building analysis pipelines and implements many convenient tools, but it comes with a complex installation procedure, heavy dependencies, and limited documentation. More importantly, it is difficult to integrate with other popular neuroimaging tools, limiting its software ecosystem.

Meanwhile, a range of versatile, interoperable open source packages for the analysis of neuroscientific data has been developed using the increasingly popular programming language Python [24]. For example, Nipy[1] is a community of practice devoted to the use of Python in the analysis of neuroimaging data, encompassing popular tools such as Nibabel [25], Nipype [26], Nilearn [27] and many others.

Here we present Nighres[2] – a new toolbox that makes the quantitative and high-resolution image processing capabilities of CBS Tools available in Python. Nighres is a user-friendly Python package which interfaces with CBS Tools while avoiding the JIST and MIPAV dependency tree. It facilitates integration with other Python-based neuroimaging tools and interactive data exploration, for example in Jupyter notebooks[3]. Nighres features comprehensive online documentation with usage examples that are based on publicly available data sets. An extensive developer's guide encourages external contributions in Java or Python. With this new package, we aim to make the capacities of CBS Tools accessible to a wider community, highlight the potential of new high-resolution image processing methods, and foster collaboration in this emerging field.

Implementation

Architecture and design

The Nighres package consists of two core Python modules. The module **cbstools** contains the original CBS Tools Java classes that have been encapsulated using the JCC package[4]. JCC encapsulates the Java code with C++ code, to make it accessible to the Python interpreter, and produces a complete Python extension module. The module **nighres** includes the Python interfaces that are exposed to the user. It is organized in submodules that represent different application areas.[5] For example, the submodule *laminar* contains functions related to laminar analysis of the cortical sheet. The Python interfaces in each submodule are currently of two types:

i. Functions that wrap Java classes
ii. Functions in pure Python

Functions that wrap Java classes

The initial motivation to develop Nighres was to provide a user-friendly interface to the functionality of CBS Tools, leveraging the flexibility of Python. Therefore, a majority of the current functions in Nighres constitute Python wrappers which internally execute the original CBS Tools Java classes. These functions generally adhere to the following basic structure (a simple example can be found in the function *probability_to_levelset*):

i. Evaluate input parameters
ii. Start Java virtual machine
iii. Initiate Java class through JCC wrapper
iv. Load input data and cast to Java array
v. Pass additional parameters to Java class
vi. Execute Java class
vii. Collect outputs of Java class and cast back
viii. Return outputs (optional: save outputs)

Thus, the actual processing still relies on the same optimized Java code as in the original CBS Tools. However, since the Nighres function takes care of the interfacing between Python and Java, the user only interacts with Python code.

Functions in pure Python

Our long-term vision is for Nighres to become a central platform for new high-resolution image processing tools as they are developed. As discussed above, Python is rapidly becoming the most popular programming language in the neuroimaging community. The modular design of Nighres allows for easy integration of pure Python processing routines, and for the use of other neuroimaging software that has been (or can be) wrapped in Python independently with pipelining tools such as Nipype [26]. In addition, we have included a core set of lightweight convenience functions for input and output, parameter handling, and file naming in Python to simplify function calls and minimize the integration burden for new methods.

1 http://nipy.org/
2 **N**euro**I**maging**G** at **H**igh **RES**olution
3 http://jupyter.org/
4 http://lucene.apache.org/pylucene/jcc/index.html
5 For consistency the submodule names are based on the original module organization in CBS Tools

Data handling

Data handling within Nighres follows established and widely used standards in the imaging community to ensure maximum interoperability. Where possible, Nighres uses the Nibabel package for handling imaging data [25]. Input and output functions are designed to automatically recognize and load most commonly used data formats, while maintaining flexibility to accommodate loading of non-standard data formats using custom scripts. Data is internally represented as Nibabel *Nifti1Images* (volumes) or Python dictionaries (surfaces) and can be passed in the form of file names or memory objects. Processing results are returned as memory objects, functions with multiple outputs return a dictionary storing the different outputs. Outputs can also be saved to disk. For saving, modifiers are appended to the output file names that refer to the name of the function and the specific output (e.g. *_layering_depth* for the continuous depth output of the volumetric layering function). Output names can be set to have a specific prefix or, by default, append modifiers to the main input file name.

Distribution

While both Python and Java are cross-platform languages, the JCC package that is used to encapsulate the CBS Tools Java classes generates C++ code and thus makes compilation platform-specific. We therefore implemented an automated build script that compiles the original CBS Tools Java code and builds the wrappers using JCC. We set up continuous integration using Travis CI[6] to test the build upon any changes to the code base on Github and, for any tagged releases, deploy the package to the Python Package Index[7]. The user can then download the package, run the fully automated build script to recompile the Java code and C++ wrappers on their platform, and finally use the pip installer[8] to install the modules and all their dependencies. Subsequently, Nighres can simply be imported into any Python environment.

We also provide a container allowing users to test Nighres in a preset environment, without actually installing it on their system. For this option the user only has to install Docker[9], a lightweight container platform that runs on Linux, Windows and Mac OS X. The Nighres Dockerfile[10] can then be used to build an Ubuntu 14 Trusty Docker image that contains a suitable Java installation, Nighres, and Jupyter Notebook.

Dependencies

One goal of Nighres was to reduce external dependencies. We therefore restricted the required packages for Nighres' core functionality to Nibabel, for reading and writing of common neuroimaging data formats [25], and Numpy, for efficient manipulation of data arrays [28]. The functions wrapping CBS Tools code require the CBS Tools Java library as well the Java matrix manipulation[11] and Apache Commons Math[12] libraries. However, these libraries are automatically recompiled, wrapped and installed from the CBS Tools github repository[13] upon installation of Nighres. Our example workflows use Nilearn's [27] plotting functionality for visualizing their results, but will automatically skip plotting if Nilearn is not installed.

6 https://travis-ci.org/nighres
7 https://pypi.python.org/pypi/nighres
8 https://pip.pypa.io/en/stable/
9 https://www.docker.com/
10 https://github.com/nighres/nighres/blob/master/Dockerfile
11 http://math.nist.gov/javanumerics/jama/
12 http://commons.apache.org/proper/commons-math/
13 https://github.com/piloubazin/cbstools-public

Support files

Nighres automatically installs all essential support files including statistical atlases for brain segmentation, look-up tables for topological constraints, templates for high-resolution spatial normalization, and a cerebellar lobular atlas [29]. In addition, example data from publicly released 7 Tesla data sets is hosted on the Nighres project page[14] at the neuroimaging informatics tools and resources clearinghouse [NITRC, 30], and automatically downloaded when running the example workflows (see below).

Documentation

Beyond functional code, clear and concise documentation is one of the most important drivers of software use and longevity. Nighres' online documentation[15] was implemented using the Sphinx documentation tool[16] and automatically generates online content from the original function docstrings, which are written according to the the Numpy/Scipy documentation guidelines[17]. This design ensures that the documentation stays up-to-date with minimal overhead for developers, and is intuitive for users. Extensive example workflows provide users with easily understandable and reproducible code, as described in the following section. Finally, the online documentation contains an in-depth developer's guide that leads contributors through all steps necessary to submit code changes, new Python functions, new wrappers for CBS Tools functions or improvements of the documentation, to the Nighres github repository. We aimed to write a guide that makes it feasible for any researcher working with high-resolution neuroimaging data to contribute to Nighres, even without much previous experience in software development.

Usage example

In the following we present one of Nighres' usage example pipelines. The example shows how to obtain a tissue classification from MP2RAGE data [31] by performing the following steps:

i. Downloading the open MP2RAGE data set from NITRC
ii. Removing the skull and creating a brain mask
iii. Atlas-guided tissue classification using a multiple object geometric deformable model (MGDM) [32]

The outputs of the plotting functions are shown in Figure 1.

Import and download

First we import `nighres` and the `os` module to set the output directory.

```
import nighres
import os

out_dir = os.path.join(os.getcwd(),'nighres_examples/
    ↪ tissue_classification')
```

We also try to import Nilearn plotting functions. If Nilearn is not installed, plotting will be skipped.

```
skip_plots = False
```

14 https://www.nitrc.org/projects/nighres/
15 http://nighres.readthedocs.io/en/latest/
16 http://www.sphinx-doc.org/en/stable/
17 https://numpydoc.readthedocs.io/en/latest/format.html

```
try:
    from nilearn import plotting
except ImportError:
    skip_plots = True
    print('Nilearn could not be imported, plotting will be
        ↪ skipped')
```

Now we download an example MP2RAGE [31] dataset that is hosted on NITRC [30]. It is the structural scan of the first subject, first session of the 7 Tesla Test-Retest dataset published by Gorgolewski et al. [18]

```
dataset = nighres.data.download_7T_TRT(out_dir)
```

Skull stripping

The first processing step is skull stripping. Only the second inversion image of the MP2RAGE sequence is required to calculate the brain mask. But if we input the quantitative T1 map and the T1-weighted image as well, they will be masked for us. We also save the outputs in the `out_dir` specified above and use a subject ID as the base file name.

```
skullstripping_results =
nighres.brain.mp2rage_skullstripping(second_inversion=
    ↪ dataset['inv2'],t1_weighted=dataset['t1w'],t1_map=
    ↪ dataset['t1map'], save_data=True,file_name='
    ↪ sub001_sess1',output_dir=out_dir)
```

To check if the skull stripping worked well ,we plot the brain mask on top of the original image (Figure 1a). Nighres, like Nilearn [27], uses Nibabel [25] *Nifti1Image* objects to pass data internally. Therefore, we can directly pass the outputs to Nilearn's plotting functions without saving and reloading. Alternatively, the images stored in `out_dir` can be opened in any common interactive viewer that can read the Nifti data format.

```
if not skip_plots:
    plotting.plot_roi(skullstripping_results['brain_mask'],
        ↪ dataset['t1w'], annotate=False, black_bg=False
        ↪ , draw_cross=False, cmap='autumn')
```

MGDM classification

Next, we use the masked data as input for tissue classification with the MGDM algorithm [32]. MGDM works with a single contrast, but can be improved with additional contrasts. In this case we use the T1-weigthed image as well as the quantitative T1 map.

```
mgdm_results = nighres.brain.mgdm_segmentation(
contrast_image1=skullstripping_results['t1w_masked'],
    ↪ contrast_type1="Mp2rage7T",contrast_image2=
    ↪ skullstripping_results['t1map_masked'],
    ↪ contrast_type2="T1map7T",save_data=True, file_name
    ↪ ="sub001_sess1", output_dir=out_dir)
```

Now we look at the topology-constrained segmentation that MGDM created (Figure 1b)

```
if not skip_plots:
    plotting.plot_img(mgdm_results['segmentation'], vmin=1,
        ↪ vmax=50, cmap='cubehelix', colorbar=True,
        ↪ annotate=False, draw_cross=False)
```

MGDM also creates an image which represents for each voxel the distance to its nearest border (Figure 1c). It is useful to assess where partial volume effects may occur.

```
if not skip_plots:
    plotting.plot_anat(mgdm_results['distance'], vmin=0,
        ↪ vmax=20, annotate=False, draw_cross=False,
        ↪ colorbar=True)
```

This examples implements a complete workflow for advanced processing of a quantitative MR contrast at high spatial resolution (voxel size = 0.5 mm isotropic). With the openly available and automatically downloaded data, any user can try out Nighres' functionality immediately after installation and then adapt the clearly explained code for their own use case.

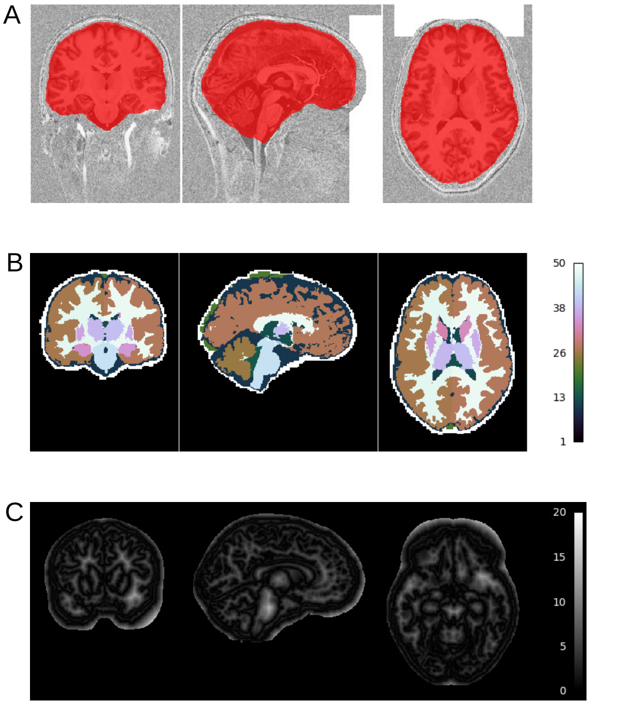

Figure 1. Tissue classification from MP2RAGE data. **A** The brain mask obtained from skull stripping. (Note that the white rectangles in the image occur because the data has been "defaced" for anonymization) **B** The segmented brain structures. **C** A representation of each voxel's distance to their nearest borders for assessing partial volume effects. Visualization performed within the script using Nilearn [27].

Discussion

We developed a Python toolbox that specializes in processing high-resolution brain imaging data. It has been designed with two key purposes in mind:

i. to provide the neuroimaging community with user-friendly access to cutting-edge high-resolution image processing tools,
ii. to create a flexible framework that can be extended by other researchers, along with thorough instructions on how to contribute.

The availability of high-resolution and quantitative MRI data, and the interest in new research directions that this data enables, are rapidly growing [e.g 33, 34]. At the same time, the image processing tools that would be required to leverage the new level of spatial detail provided by this data are largely missing. Only a few major neuroimaging packages have begun to adapt their tools for these purposes [35, 36]. However, these packages are limited by their closed source

code or rigid data organization, while it is crucial that newly emerging methods can be flexibly adapted, collaboratively developed, and integrated with other tools.

CBS Tools provides dedicated open source methods for high-resolution image processing [20]. Unfortunately, its complex design and heavy dependencies can make the installation and handling challenging for naive users, and impede contributions from other researchers. With Nighres we provide a flexible and user-friendly implementation of CBS Tools' functionality, which eliminates the dependency on MIPAV and JIST. Another major advance of Nighres compared to CBS Tools is its extensive online documentation. Besides comprehensives explanation of every function's in- and outputs, carefully documented usage examples provide step-by-step instructions of how the different tools can be combined to create complete processing pipelines.

The current implementation of Nighres contains a set of cutting-edge methods, but rapid methodological advances are to be expected in the dynamic field of high-resolution neuroimaging. We therefore designed Nighres as a transparent software platform through which newly developed methods can be made available to the community and improved collaboratively. New or existing tools can easily be added in a variety of formats, depending on the specific requirements of the operation and the preferences of the developer. The extensive developer's guide aims to encourage contributions even from researchers with little previous experience in software development.

We aimed to closely integrate our package with the existing community around neuroimaging tools in Python. To this end, we adopted standardized objects for internal data handling, which can easily be exchanged with other tools. An example is the seamless visualization of Nighres outputs using Nilearn's [27] plotting functions as showcased in the usage example (Figure 1).

A major limitation of the current package is that it has been developed and tested for common Linux platforms only. The C++ code generated by JCC to interfaces with the CBS Tools Java classes makes the compilation platform dependent. We addressed this issue by providing an automated build script that recompiles this code upon installation. While this process has only been tested on Linux, the design makes a future adaptation to Mac OS X platforms straightforward. Support for Windows is not currently planned. However, the provided Dockerfile enables usage of Nighres in a container on any platform that supports Docker.

Many future extension of the current package can be envisioned. Besides integrating more of the original CBS Tools functions, a main goal is to extend functionality with new tools coded directly in Python. To ensure efficient processing of the large data this might require the implementation of critical processes as C-extension through Cython[18]. Another goal is to provide integration with tools for parallel processing and job management on compute clusters.

In sum, we developed a user-friendly and well-documented Python package that makes cutting-edge high-resolution image processing tools available to the research community. The toolbox is easy to install and provides a comprehensive set of advanced techniques. While the current functionality is largely based on CBS Tools, we hope that the flexible framework encourages contribution of new tools, stimulates collaboration, and accelerates progress in the promising field of high-resolution neuroimaging.

18 http://cython.org/

Availability and requirements

- Project name: Nighres
- Project home page: https://github.com/nighres/nighres
- Operating system(s): Linux
- Programming language: Python, Java
- Other requirements: Java≥1.7, Python≥2.7, Numpy≥1.13, Nibabel≥2.1.0
- License: Apache License 2.0

Availability of supporting data

The data sets supporting the results of this article are available in the NITRC image repository [30] under https://www.nitrc.org/frs/?group_id=1205.

Declarations

List of abbreviations

- MGDM – multiple object geometric deformable model
- MPM – quantitative multi-parameter mapping
- MP2RAGE – magnetization prepared two rapid acquisition gradient echoes
- MRI – magnetic resonance imaging
- NITRC – the neuroimaging informatics tools and resources clearinghouse
- QSM – quantitative susceptibility mapping

Ethical Approval

Not applicable.

Consent for publication

Not applicable.

Competing Interests

The authors declare that they have no competing interests.

Funding

JMH project was partially funded by a stipend from Google via the Google Summer of Code program, with INCF as mentoring organization.

Author's Contributions

JMH, CJS and PLB contributed equally to conceptualization of the project and writing of the manuscript. JMH lead and CJS and PLB supported software development. All authors read and approved the final manuscript.

Acknowledgements

We would like to thank Gilles de Hollander, Nathaniel Kofalt and Rüdiger Meier for their contributions to Nighres, Daniel S. Margulies for his continuous support of this project, and Malin Sandström and the INCF for their coordination of the Google Summer of Code project.

References

1. van der Zwaag W, Schäfer A, Marques JP, Turner R, Trampel R. Recent applications of UHF-MRI in the study of human brain function and structure: a review: UHF MRI: Applications to Human Brain Function and Structure. NMR in Biomedicine 2016 Sep;29(9):1274–1288. http://doi.wiley.com/10.1002/nbm.3275.
2. Lutti A, Dick F, Sereno MI, Weiskopf N. Using high-resolution quantitative mapping of R1 as an index of cortical myelination. NeuroImage 2014 Jun;93:176–188. http://linkinghub.elsevier.com/retrieve/pii/S1053811913006423.
3. Sereno MI, Lutti A, Weiskopf N, Dick F. Mapping the human cortical surface by combining quantitative T1 with retinotopy. Cereb Cortex 2013;23(9):2261–2268.
4. Dick F, Tierney AT, Lutti A, Josephs O, Sereno MI, Weiskopf N. In vivo functional and myeloarchitectonic mapping of human primary auditory areas. J Neurosci 2012;32(46):16095–16105.
5. Huntenburg JM, Bazin PL, Goulas A, Tardif CL, Villringer A, Margulies DS. A Systematic Relationship Between Functional Connectivity and Intracortical Myelin in the Human Cerebral Cortex. Cerebral Cortex 2017 Feb;27(2):981–997. https://academic.oup.com/cercor/article/2981936/A.
6. Dinse J, Härtwich N, Waehnert MD, Tardif CL, Schäfer A, Geyer S, et al. A cytoarchitecture-driven myelin model reveals area-specific signatures in human primary and secondary areas using ultra-high resolution in-vivo brain MRI. NeuroImage 2015 Jul;114:71–87. http://linkinghub.elsevier.com/retrieve/pii/S1053811915003134.
7. Waehnert MD, Dinse J, Schäfer A, Geyer S, Bazin PL, Turner R, et al. A subject-specific framework for in vivo myeloarchitectonic analysis using high resolution quantitative MRI. NeuroImage 2016 Jan;125:94–107. http://linkinghub.elsevier.com/retrieve/pii/S1053811915008897.
8. Fracasso A, van Veluw SJ, Visser F, Luijten PR, Spliet W, Zwanenburg JJM, et al. Lines of Baillarger in vivo and ex vivo: Myelin contrast across lamina at 7T MRI and histology. Neuroimage 2016;133:163–175.
9. Whitaker KJ, Vértes PE, Romero-Garcia R, Váša F, Moutoussis M, Prabhu G, et al. Adolescence is associated with genomically patterned consolidation of the hubs of the human brain connectome. Proc Natl Acad Sci U S A 2016;113(32):9105–9110.
10. Marques JP, Khabipova D, Gruetter R. Studying cyto and myeloarchitecture of the human cortex at ultra-high field with quantitative imaging: R1, R2(*) and magnetic susceptibility. Neuroimage 2017;147:152–163.
11. Kok P, Bains L, vanMourik T, Norris D, deLange F. Selective Activation of the Deep Layers of the Human Primary Visual Cortex by Top-Down Feedback. Current Biology 2016 Feb;26(3):371–376. http://linkinghub.elsevier.com/retrieve/pii/S0960982215015699.
12. Huber L, Handwerker D, Gonzalez-Castillo J, Jangraw MS D, Guidi M, et al, Directional connectivity measured with layer-dependent fMRI in human sensory-motor system;. Talk presented at 3rd Biennial Whistler Scientific Workshop on Brain Functional Organization, Connectivity and Behavior, March 6-9, 2016, Whistler, BC, Canada.
13. Huber L, Handwerker D, Gonzalez-Castillo J, Guidi M, Ivanov D, Poser B, et al., Methods for measuring effective connectivity with high resolution blood volume fMRI;. Talk presented at 24th Annual Meeting of the International Society for Magnetic Resonance in Medicine, May 7-9, 2016, Singapore.
14. Keuken MC, Bazin PL, Crown L, Hootsmans J, Laufer A, Müller-Axt C, et al. Quantifying inter-individual anatomical variability in the subcortex using 7T structural MRI. NeuroImage 2014 Jul;94:40–46. http://linkinghub.elsevier.com/retrieve/pii/S1053811914001797.
15. Steele CJ, Anwander A, Bazin PL, Trampel R, Schaefer A, Turner R, et al. Human Cerebellar Sub-millimeter Diffusion Imaging Reveals the Motor and Non-motor Topography of the Dentate Nucleus. Cerebral Cortex 2017;27(9):4537–4548. +http://dx.doi.org/10.1093/cercor/bhw258.
16. Thürling M, Kahl F, Maderwald S, Stefanescu RM, Schlamann M, Boele HJ, et al. Cerebellar cortex and cerebellar nuclei are concomitantly activated during eyeblink conditioning: a 7T fMRI study in humans. J Neurosci 2015 Jan;35(3):1228–1239.
17. Forstmann BU, Keuken MC, Schafer A, Bazin PL, Alkemade A, Turner R. Multi-modal ultra-high resolution structural 7-Tesla MRI data repository. Sci Data 2014 Dec;1:140050.
18. Gorgolewski KJ, Mendes N, Wilfling D, Wladimirow E, Gauthier CJ, Bonnen T, et al. A high resolution 7-Tesla resting-state fMRI test-retest dataset with cognitive and physiological measures. Sci Data 2015 Jan;2:140054.
19. Tardif CL, Schäfer A, Trampel R, Villringer A, Turner R, Bazin PL. Open Science CBS Neuroimaging Repository: Sharing ultra-high-field MR images of the brain. Neuroimage 2016;124:1143–1148.
20. Bazin PL, Weiss M, Dinse J, Schäfer A, Trampel R, Turner R. A computational framework for ultra-high resolution cortical segmentation at 7Tesla. Neuroimage 2014 Jun;93 Pt 2:201–209.
21. Bazin PL, Plessis V, Fan AP, Villringer A, Gauthier CJ. Vessel segmentation from quantitative susceptibility maps for local oxygenation venography. IEEE; 2016. p. 1135–1138. http://ieeexplore.ieee.org/lpdocs/epic03/wrapper.htm?arnumber=7493466.
22. McAuliffe MJ, Lalonde FM, McGarry D, Gandler W, Csaky K, Trus BL. Medical Image Processing, Analysis and Visualization in clinical research. In: Computer-Based Medical Systems, 2001. CBMS 2001. Proceedings. 14th IEEE Symposium on; 2001. p. 381–386.
23. Lucas BC, Bogovic JA, Carass A, Bazin PL, Prince JL, Pham DL, et al. The Java Image Science Toolkit (JIST) for rapid prototyping and publishing of neuroimaging software. Neuroinformatics 2010 Mar;8(1):5–17.
24. Muller E, Bednar JA, Diesmann M, Gewaltig MO, Hines M, Davison AP. Python in neuroscience. Front Neuroinform 2015 Apr;9:11.
25. Brett M, Hanke M, Cipollini B, Côté MA, Markiewicz C, Gerhard S, et al. nibabel: 2.1. 0. Zenodo 2016;.
26. Gorgolewski KJ, Burns CD, Madison C, Clark D, Halchenko YO. Nipype : a flexible, lightweight and extensible neuroimaging data processing framework in Python. Front Neuroinform 2011;5(August).
27. Abraham A, Pedregosa F, Eickenberg M, Gervais P, Mueller A, Kossaifi J, et al. Machine learning for neuroimaging with scikit-learn. Front Neuroinform 2014;8:14.
28. van der Walt S, Colbert SC, Varoquaux G. The NumPy Array: A Structure for Efficient Numerical Computation. Computing in Science Engineering 2011 March;13(2):22–30.
29. Bazin J P L Kipping, Steele CJ, Margulies D, Turner R, Villringer A, Subject-specific cortical cerebellar mapping at 3T

and 7T;. Poster presented at the 19th Annual Meeting of the Organization for Human Brain Mapping, June 16-20, 2013, Seattle, USA.
30. Kennedy DN, Haselgrove C, Riehl J, Preuss N, Buccigrossi R. The NITRC image repository. NeuroImage 2016;124(Part B):1069–1073.
31. Marques JP, Kober T, Krueger G, van der Zwaag W, Van de Moortele PF, Gruetter R. MP2RAGE, a self bias-field corrected sequence for improved segmentation and T1-mapping at high field. Neuroimage 2010 Jan;49(2):1271–1281.
32. Bogovic J, Prince J, Bazin P. A multiple object geometric deformable model for image segmentation. Computer Vision and Image Understanding 2013;117(2):145–157.
33. Trampel R, Bazin PL, Pine K, Weiskopf N. In-vivo magnetic resonance imaging (MRI) of laminae in the human cortex. Neuroimage 2017 Sep;.
34. Paus T. Imaging microstructure in the living human brain: A viewpoint. NeuroImage 2017;http://www.sciencedirect.com/science/article/pii/S1053811917308261.
35. Goebel R. BrainVoyager–past, present, future. Neuroimage 2012 Aug;62(2):748–756.
36. Zaretskaya N, Fischl B, Reuter M, Renvall V, Polimeni JR. Advantages of cortical surface reconstruction using submillimeter 7 T MEMPRAGE. Neuroimage 2017 Sep;165:11–26.
