# Supplementary material for: Nighres: processing tools for high-resolution neuroimaging
Source: Gigascience. 2018 Jul 4;7(7):giy082. doi: 10.1093/gigascience/giy082 (PMC6065481; doi:10.1093/gigascience/giy082)
Supplement: GIGA-D-17-00325_Revision_1.pdf [file giy082_giga-d-17-00325_revision_1.pdf]

GigaScience

Nighres: Processing tools for high-resolution neuroimaging

--Manuscript Draft--

**Manuscript Number:**	GIGA-D-17-00325R1
**Full Title:**	Nighres: Processing tools for high-resolution neuroimaging
**Article Type:**	Technical Note
**Funding Information:**	
**Abstract:**	With recent improvements in human magnetic resonance imaging (MRI) at ultra-high fields, the amount of data collected per subject in a given MRI experiment has increased considerably. Standard image processing packages are often challenged by the size of these data and dedicated methods are needed to leverage their extraordinary spatial resolution. Here we introduce a flexible Python toolbox which implements a set of advanced techniques for high-resolution neuroimaging. With these tools, segmentation and laminar analysis of cortical MRI data can be performed at resolutions up to 500 μm in reasonable times. Comprehensive online documentation makes the toolbox easy to use and install. An extensive developer's guide encourages contributions of other researchers that will help to accelerate progress in the promising field of high-resolution neuroimaging.
**Corresponding Author:**	Julia M Huntenburg<br>Max-Planck-Institut fur Kognitions- und Neurowissenschaften<br>Leipzig, GERMANY
**Corresponding Author Secondary Information:**	
**Corresponding Author's Institution:**	Max-Planck-Institut fur Kognitions- und Neurowissenschaften
**Corresponding Author's Secondary Institution:**	
**First Author:**	Julia M Huntenburg
**First Author Secondary Information:**	
**Order of Authors:**	Julia M Huntenburg
	Christopher J Steele
	Pierre-Louis Bazin
**Order of Authors Secondary Information:**	
**Response to Reviewers:**	We have now included the reference to the GigaDB entry in the section "Availability of supporting data".
**Additional Information:**	
**Question**	**Response**
Are you submitting this manuscript to a special series or article collection?	No
**Experimental design and statistics**<br><br>Full details of the experimental design and statistical methods used should be given in the Methods section, as detailed in our Minimum Standards Reporting Checklist. Information essential to interpreting the data presented should be made available in the figure legends.	No

Powered by Editorial Manager® and ProduXion Manager® from Aries Systems Corporation

Have you included all the information requested in your manuscript?	
If not, please give reasons for any omissions below.<br><br>as follow-up to "**Experimental design and statistics**<br><br>Full details of the experimental design and statistical methods used should be given in the Methods section, as detailed in our Minimum Standards Reporting Checklist. Information essential to interpreting the data presented should be made available in the figure legends.<br><br>Have you included all the information requested in your manuscript?<br><br>"	Not applicable as this manuscript describes software
**Resources**<br><br>A description of all resources used, including antibodies, cell lines, animals and software tools, with enough information to allow them to be uniquely identified, should be included in the Methods section. Authors are strongly encouraged to cite Research Resource Identifiers (RRIDs) for antibodies, model organisms and tools, where possible.<br><br>Have you included the information requested as detailed in our Minimum Standards Reporting Checklist?	Yes
**Availability of data and materials**<br><br>All datasets and code on which the conclusions of the paper rely must be either included in your submission or deposited in publicly available repositories (where available and ethically appropriate), referencing such data using a unique identifier in the references and in the "Availability of Data and Materials" section of your manuscript.<br><br>Have you have met the above requirement as detailed in our Minimum Standards Reporting Checklist?	Yes

Powered by Editorial Manager® and ProduXion Manager® from Aries Systems Corporation

Main Latex file
Click here to access/download;Manuscript;main.tex
Click here to view linked References

Placeholder for OUP logo oup.pdf

GigaScience, 0000, 1–9

doi: xx.xxxx/xxxx
Manuscript in Preparation
Technical Note

TECHNICAL NOTE

Nighres: Processing tools for high-resolution neuroimaging

Julia M Huntenburg[1,2,*], Christopher J Steele[3,4,†] and Pierre-Louis Bazin[3,5,6,7,†]

[1]Max Planck Research Group for Neuroanatomy & Connectivity, Max Planck Institute for Human Cognitive and Brain Sciences, Leipzig, Germany and [2]Neurocomputation and Neuroimaging Unit, Department of Education and Psychology, Free University of Berlin, Berlin, Germany and [3]Department of Neurology, Max Planck Institute for Human Cognitive and Brain Sciences, Germany and [4]Cerebral Imaging Center, Douglas Mental Health University Institute, Montreal, QC, Canada and [5]Department of Neurophysics, Max Planck Institute for Human Cognitive and Brain Sciences, Leipzig, Germany and [6]Social Brain Lab, Netherlands Institute for Neuroscience, Amsterdam, Netherlands and [7]Spinoza Centre for Neuroimaging, Amsterdam, Netherlands

[*]Correspondance: ju.huntenburg@gmail.com
[†]Contributed equally

Abstract

With recent improvements in human magnetic resonance imaging (MRI) at ultra-high fields, the amount of data collected per subject in a given MRI experiment has increased considerably. Standard image processing packages are often challenged by the size of these data and dedicated methods are needed to leverage their extraordinary spatial resolution. Here we introduce a flexible Python toolbox which implements a set of advanced techniques for high-resolution neuroimaging. With these tools, segmentation and laminar analysis of cortical MRI data can be performed at resolutions up to 500 μm in reasonable times. Comprehensive online documentation makes the toolbox easy to use and install. An extensive developer's guide encourages contributions of other researchers that will help to accelerate progress in the promising field of high-resolution neuroimaging.

Key words: Neuroimaging in Python; High-resolution MRI; Ultra-high field MRI; Laminar MRI; Python Java integration

Background

Advances in ultra-high field (7 Tesla and above) MRI now make it possible to image the entire human brain at an unprecedented level of detail [1]. Submillimeter resolutions and quantitative metrics reveal fine-grained variations in structure and function that were previously undetectable in vivo. This information allows researchers to ask new questions about the human brain. Examples include the investigation of intracortical myelin [e.g 2, 3, 4, 5], the laminar organization of the cortical sheet [e.g. 6, 7, 8, 9, 10], feedforward and feedback patterns in cortical connections [11, 12] and the detailed description of small cortical and subcortical structures [13, 14] and their function [15].

While ultra-high field scanners have become increasingly available, and the first open 7 Tesla (T) MRI data sets have been released [16, 17, 18], software tools still lag behind. Standard neuroimaging software packages are often not designed to handle the growing data size and new quantitative contrasts. Three-dimensional MRI data grows as a cube of its resolution, and computational complexity generally

Compiled on: June 26, 2018.
Draft manuscript prepared by the author.

Key Points

- A toolbox dedicated to the processing of high-resolution MRI data
- Lightweight and flexible code written in Python for ease of use, expansion and integration with other tools
- Extensive documentation with developer's guide and usage examples based on open data

ranges between $O(N \log N)$ and $O(N^2)$. Therefore, a change in spatial resolution from 1 mm to 0.5 mm easily entails an increase in computational requirements by a factor of 15 to 60, depending on the methods used. Moreover, new applications such as laminar analysis have only become possible with higher resolutions and are not implemented in many existing software packages.

CBS High-Res Brain Processing Tools (CBS Tools) is a software suite which addresses this gap by providing cutting-edge methods for efficient processing of MR images at submillimeter resolution [19]. For example, CBS Tools implements routine cortical segmentation at resolutions as high as 400 μm, processing of quantitative MRI sequences such as MP2RAGE, MPM or QSM [19], laminar analysis [7], and small vessel segmentation [20]. While this software has been well-received as a key tool set for quantitative and high-resolution neuroimaging, its adoption has been slowed by the complex infrastructure it builds on. CBS Tools have been developed in Java as a set of plugins for the MIPAV software package [21] and the JIST pipeline environment [22]. The MIPAV and JIST framework provides a graphical interface for building analysis pipelines and implements many convenient tools, but it comes with a complex installation procedure, heavy dependencies, and limited documentation. More importantly, it is difficult to integrate with other popular neuroimaging tools, limiting its software ecosystem.

Meanwhile, a range of versatile, interoperable open source packages for the analysis of neuroscientific data has been developed using the increasingly popular programming language Python [23]. For example, Nipy[1] is a community of practice devoted to the use of Python in the analysis of neuroimaging data, encompassing popular tools such as Nibabel [24], Nipype [25], Nilearn [26] and many others.

Here we present Nighres[2] – a new toolbox that makes the quantitative and high-resolution image processing capabilities of CBS Tools available in Python. Nighres is a user-friendly Python package which interfaces with CBS Tools while avoiding the JIST and MIPAV dependency tree. It facilitates integration with other Python-based neuroimaging tools and interactive data exploration, for example in Jupyter notebooks.[3] Nighres features comprehensive online documentation with usage examples that are based on publicly available data sets. An extensive developer's guide encourages external contributions. With this new package, we aim to make the functionality of CBS Tools accessible to a wider community, highlight the potential of new high-resolution image processing methods, and foster collaboration in this emerging field.

1 http://nipy.org/
2 **N**euro**I**maginG at **H**igh **RES**olution
3 http://jupyter.org/

Implementation

Architecture and design

The Nighres package consists of two core Python modules. The module **cbstools** contains the original CBS Tools Java classes that have been encapsulated using the JCC package.[4] JCC encapsulates the Java code with C++ code, to make it accessible to the Python interpreter, and produces a complete Python extension module. The module **nighres** includes the Python interfaces that are exposed to the user. It is organized in submodules that represent different application areas.[5] For example, the submodule *laminar* contains functions related to laminar analysis of the cortical sheet. There are currently two types of Python interfaces within these submodules:

i. Functions that wrap Java classes
ii. Functions in pure Python

Functions that wrap Java classes
The initial motivation to develop Nighres was to provide a user-friendly interface to the functionality of CBS Tools, leveraging the flexibility of Python. Therefore, a majority of the current functions in Nighres constitute Python wrappers which internally execute the original CBS Tools Java classes. These functions generally adhere to the following basic structure (a simple example can be found in the function *probability_to_levelset*):

i. Evaluate input parameters
ii. Start Java virtual machine
iii. Initiate Java class through JCC wrapper
iv. Load input data and cast to Java array
v. Pass additional parameters to Java class
vi. Execute Java class
vii. Collect outputs of Java class and cast back
viii. Return outputs (optional: save outputs)

Thus, the actual processing still relies on the same optimized Java code as in the original CBS Tools. However, since the Nighres function takes care of the interfacing between Python and Java, the user *only* interacts with Python code.

Functions in pure Python
Our long-term vision is for Nighres to become a central platform for new high-resolution image processing tools as they are developed. As discussed above, Python is rapidly becoming the most popular programming language in the neuroimaging community [23]. The modular design of Nighres allows for easy integration of pure Python processing routines, meaning that new functions can be contributed without the need to interact with Java or to learn about the JCC based wrapping procedure. In addition, it is possible to integrate useful tools from other neuroimaging software that have been (or can be) wrapped in Python for example using Nipype [25]. Currently, Nighres in-

4 http://lucene.apache.org/pylucene/jcc/index.html
5 For consistency the submodule names are based on the original module organization in CBS Tools

cludes a core set of Python functions for input and output, parameter handling, and file naming to simplify function calls and minimize the integration burden for new methods.

Data handling

Data handling within Nighres follows established and widely used standards in the neuroimaging community to ensure maximal interoperability. Where possible, Nighres uses the Nibabel package for handling imaging data [24]. Input and output functions are designed to automatically recognize and load most commonly used data formats, while maintaining flexibility to accommodate loading of non-standard data formats using custom scripts. Data is internally represented as Nibabel *Nifti1Images* (volumes) or Python dictionaries (surfaces) and can be passed in the form of file names or memory objects. Processing results are returned as memory objects, functions with multiple outputs return a dictionary storing the different outputs. Outputs can also be saved to disk. For saving, modifiers are appended to the output file names that refer to the name of the function and the specific output (e.g. *_layering_depth* for the continuous depth output of the layering function). Output names can be set to have a specific prefix or, by default, append modifiers to the main input file name.

Distribution

While both Python and Java are cross-platform languages, the JCC package encapsulates CBS Tools' Java classes with C++ code and thus makes the compilation platform-specific. We implemented an automated build script that compiles the original CBS Tools Java code and builds the wrappers using JCC. We set up continuous integration using Travis CI[6] to test the build upon any changes to the code base on Github and, for any tagged releases, deploy the package to the Python Package Index.[7] The user can then download the package, run the fully automated build script to recompile the Java code and C++ wrappers on their platform, and finally use the pip installer[8] to install the modules and all their dependencies. Subsequently, Nighres can simply be imported into any Python environment. We also provide a container allowing users to test Nighres in a preset environment, without actually installing it on their system. For this option the user only has to install Docker,[9] a lightweight container platform that runs on Linux, Windows and Mac OS X. The Nighres Dockerfile[10] can then be used to build an Ubuntu 14 Trusty Docker image that contains a suitable Java installation, Nighres, and Jupyter Notebook.

Dependencies

One goal of Nighres was to reduce external dependencies. We therefore restricted the required packages for Nighres' core functionality to Nibabel, for reading and writing of common neuroimaging data formats [24], and Numpy, for efficient manipulation of data arrays [27]. The functions wrapping CBS Tools code require the CBS Tools Java library as well the Java matrix manipulation[11] and Apache Commons Math[12] libraries. However, these libraries are automatically recompiled, wrapped and installed from the CBS Tools github repository[13] upon installation of Nighres. Our example workflows use Nilearn's [26] plotting functionality for visualizing their results, but will automatically skip plotting if Nilearn is not installed.

Support files

Nighres automatically installs all essential support files including statistical atlases for brain segmentation, look-up tables for topological constraints, templates for high-resolution spatial normalization, and a cerebellar lobular atlas [28]. Example data from publicly released 7T data sets is hosted on the Nighres project page[14] at the neuroimaging informatics tools and resources clearinghouse [NITRC, 29]. This data is automatically downloaded when running the example workflows.

Documentation

Beyond functional code, clear and concise documentation is one of the most important drivers of software use and longevity. Nighres' online documentation[15] was implemented using the Sphinx documentation tool.[16] The online content is automatically generated from the original function docstrings, which are written according to the the Numpy/Scipy documentation guidelines.[17] This design ensures that the documentation stays up-to-date with minimal overhead for developers, and is intuitive for users. Extensive example workflows provide users with easily understandable and reproducible code (see section *Usage example* below). Finally, the online documentation contains an in-depth developer's guide that leads contributors through all steps necessary to submit code changes, new Python functions or CBS Tools wrappers to the Nighres github repository. We aimed to write a guide that makes it feasible for any researcher working with high-resolution neuroimaging data to contribute to Nighres, even without much previous experience in software development.

Functionality

Nighres contains a set of advanced functions which are not commonly implemented in neuroimaging software and/or have been optimized towards the specific demands of processing high-resolution and quantitative neuroimaging data. In this section, we provide an overview of the major features that are currently implemented. Their application will be demonstrated in the subsequent section, which also indicates example computation times. A more in-depth discussion of the individual algorithms and their performance can be found in the original references listed for each function.

MP2RAGE skull-stripping

This fast skull-stripping algorithm has been optimized for quantitative images acquired at 7T using the MP2RAGE sequence [30]. See [19] for details.

Multiple object geometric deformable model segmentation (MGDM)

MGDM is a whole brain tissue classification method, designed to routinely process datasets at resolutions up to 400 μm. A variety of inputs (MP2RAGE at 3T, 7T and 9.4T; MPM at 3T and 7T; T1-, T2- and diffusion-weighted images) as well as multiple inputs are accepted. This atlas-guided method uniquely

6 https://travis-ci.org/nighres
7 https://pypi.python.org/pypi/nighres
8 https://pip.pypa.io/en/stable/
9 https://www.docker.com/
10 https://github.com/nighres/nighres/blob/master/Dockerfile
11 http://math.nist.gov/javanumerics/jama/
12 http://commons.apache.org/proper/commons-math/
13 https://github.com/piloubazin/cbstools-public
14 https://www.nitrc.org/projects/nighres/
15 http://nighres.readthedocs.io/en/latest/
16 http://www.sphinx-doc.org/en/stable/
17 https://numpydoc.readthedocs.io/en/latest/format.html

preserves the topological properties and relationships of all 25 classified brain structures. See [19, 31, 32] for details.

Cortical reconstruction using implicit surface evolution (CRUISE)
CRUISE provides a precise and efficient method to extract cortical surfaces from high-resolution volumetric data based on level set representations (see next subsection). A distinguishing feature of this algorithm is the careful modeling of sulcal fundi. CRUISE can be applied to cerebral and cerebellar cortices and to data with partial brain coverage. See [33] for details.

Level set creation
This function creates level sets from probabilistic or deterministic tissue classifications. Level sets are signed distance functions that can be used for representing cortical surfaces in voxel space instead of triangular meshes. Such representations have favorable mathematical properties, avoid mesh sampling problems and facilitate the integration of volumetric and surface data. Several Nighres functions rely on level sets internally. See [19] for details.

Equivolumetric layering
Nighres implements an equivolumetric technique for modeling intracortical laminae. This approach accounts for the dependency of layer thickness on local curvature by preserving the volume of cortical segments [cf. 34]. The resulting cortical depth estimates represent an intracortical coordinate system that is anatomically more accurate than commonly applied equidistant or Laplacian approaches. Intracortical surfaces are represented as level sets. See [35] for details.

In addition to the aforementioned functions, we are currently preparing to migrate CBS Tools' multi-modal surface registration algorithm [36] and non-linear deformation utilities, as well as algorithms for topology correction [37] and vascular segmentation [20] into the Nighres package.

Usage example

In the following we present a Nighres usage example. It shows how to obtain a tissue classification and cortical depth estimation from MP2RAGE data, acquired at 7T with a resolution of 0.7 mm isotropic. The pipeline contains the following steps:

i. Downloading the open MP2RAGE data set from NITRC
ii. Removing the skull and creating a brain mask
iii. Atlas-guided tissue classification using MGDM [31]
iv. Extracting the cortex of one hemisphere
v. Cortical reconstruction using CRUISE [33]
vi. Equivolumetric modeling of intracortical laminae [35]

The outputs of the plotting functions are shown in Figure 1 and 2. Average computation times for the different processing steps in this example are indicated in Table 1. They were determined on a standard laptop (8GB RAM, i7-5500U dual core processor, 4 MB cache, 3 GHz maximum frequency) using Python's `timeit` module.[18]

Import and download

First, we import `nighres` and the `os` module to set the output directory.

```
import nighres, os
```

```
out_dir = os.path.join(os.getcwd(),
            "nighres_examples/tissue_classification")
```

We also import Nilearn's plotting functions. If Nilearn is not installed, plotting will be skipped in the online examples.

```
from nilearn import plotting
```

Now we download an example MP2RAGE dataset that is hosted on NITRC. It is the structural scan of the first subject, first session of the 7T test-retest dataset published in [17].

```
dataset = nighres.data.download_7T_TRT(out_dir)
```

Skull stripping

The first processing step is skull stripping. The brain mask is calculated based on the second inversion image of the MP2RAGE sequence. For convenience, we can also input the quantitative T1 map and the T1-weighted image, to which the calculated brain mask will then be applied. We save the outputs in the `out_dir` specified above and use a subject ID as the base file name.

```
skullstrip_results = nighres.brain.mp2rage_skullstripping(
        second_inversion=dataset["inv2"],
        t1_weighted=dataset["t1w"],
        t1_map=dataset["t1map"],
        save_data=True, output_dir=out_dir
        file_name="sub001_sess1")
```

To check if the skull stripping worked well, we plot the brain mask on top of the original image (Figure 1a). Nighres, like Nilearn, uses Nibabel's *Nifti1Image* object to pass data internally. Therefore, we can directly pass the outputs to Nilearn's plotting functions without saving and reloading. Alternatively, the images stored in `out_dir` can be opened in any common interactive viewer that can read the Nifti data format.

```
plotting.plot_roi(skullstrip_results["brain_mask"],
        dataset["t1w"], cut_coords=[15, 25, 30],
        annotate=False, black_bg=False, draw_cross=False,
        cmap="PuRd_r")
```

(We hereafter omit the plotting code, it can be found in the online documentation).

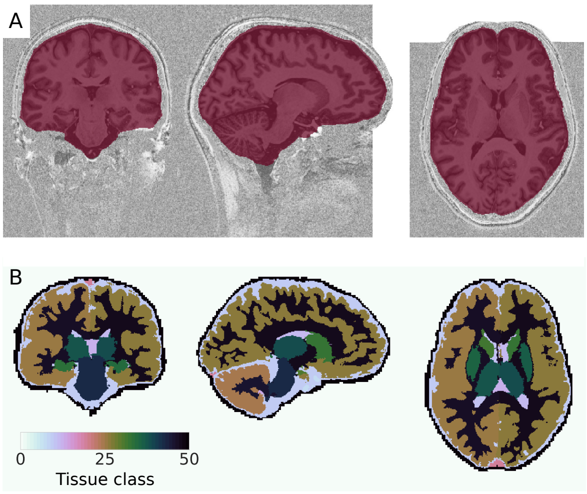

Figure 1. Tissue classification from MP2RAGE data. A The brain mask obtained from skull stripping. (Note that the white rectangles in the image occur because the data has been "defaced" for anonymization) **B** The result of the MGDM tissue classification. Visualized using Nilearn [26].

18 https://docs.python.org/2/library/timeit.html

MGDM tissue classification

Next, we use the masked data as input for tissue classification with the MGDM algorithm [31]. MGDM works with a single contrast, but can be improved with additional contrasts. In this case we use the T1-weighted image as well as the quantitative T1 map.

```
mgdm_results = nighres.brain.mgdm_segmentation(
        contrast_image1=skullstrip_results["t1w_masked"],
        contrast_type1="Mp2rage7T",
        contrast_image2=skullstrip_results["t1map_masked"],
        contrast_type2="T1map7T",
        save_data=True, output_dir=out_dir,
        file_name="sub001_sess1")
```

The topology-constrained segmentation that MGDM creates is shown in Figure 1b.

Cortical surface reconstruction

First we extract from the MGDM output the regions needed for cortical reconstruction. The outputs are membership functions for the following regions: the gray matter cortex ('region'), the underlying white matter (with filled subcortex and ventricles, 'inside') and the surrounding cerebrospinal fluid (with masked regions, 'background').

```
cortex = nighres.brain.extract_brain_region(
        segmentation=mgdm_results["segmentation"],
        levelset_boundary=mgdm_results["distance"],
        maximum_membership=mgdm_results["memberships"],
        maximum_label=mgdm_results["labels"],
        extracted_region="left_cerebrum",
        save_data=True, output_dir=out_dir,
        file_name="sub001_sess1_left_cerebrum")
```

Next, we use the extracted data as input for cortical reconstruction with the CRUISE algorithm [33]. CRUISE uses the membership functions as a guide and the white matter mask as a (topologically spherical) starting point to grow refined boundaries between the gray and white matter and the gray matter and the cerebrospinal fluid.

```
cruise = nighres.cortex.cruise_cortex_extraction(
        init_image=cortex["inside_mask"],
        wm_image=cortex["inside_proba"],
        gm_image=cortex["region_proba"],
        csf_image=cortex["background_proba"],
        normalize_probabilities=True,
        save_data=True, output_dir=out_dir
        file_name="sub001_sess1_left_cerebrum")
```

The topology-constrained segmentation with refined boundaries that CRUISE created is shown in Figure 2a.

Modeling of intracortical laminae

Finally, we use the gray-white matter boundary (GWB) and cerebrospinal fluid-gray matter boundary (CGB) from CRUISE to compute cortical depth and model intracortical laminae. Importantly, the equivolumetric approach implemented in Nighres accounts for the dependency of layer thickness on cortical folding [for an in-depth discussion see 35].

```
depth = nighres.laminar.volumetric_layering(
        inner_levelset=cruise["gwb"],
        outer_levelset=cruise["cgb"],
        n_layers=4,
        save_data=True, output_dir=out_dir,
        file_name="sub001_sess1_left_cerebrum")
```

Figure 2b shows the continuous equivolumetric depth estimate. The function call also outputs discrete representations of the modeled laminae as well as level sets describing the intracortical surfaces.

In summary, this example implements a complete workflow for advanced processing of a quantitative MR contrast at high spatial resolution (voxel size = 0.7 mm isotropic). With the openly available and automatically downloaded data, any user can try out Nighres' functionality immediately after installation and then adapt the code for their own use case. The examples can be found in our online documentation,[19] where the code can be downloaded as Python scripts or Jupyter notebooks.

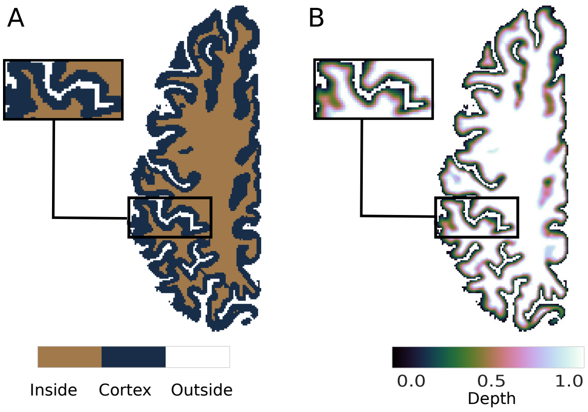

Figure 2. Cortical surface reconstruction and depth estimation. A Topology-constrained reconstruction of the boundaries between the cortical grey matter (*Cortex, blue*) the cerebrospinal fluid (*Outside, white*) and the white matter (*Inside, brown*) using CRUISE [33] **B** Intracortical depth estimated using an equivolumetric approach [35]. Visualized using Nilearn [26].

Discussion

The availability of high-resolution and quantitative MRI data and the interest in new research directions that this data enables are rapidly growing [e.g 38, 39]. At the same time, image processing tools required to leverage the new level of spatial detail provided by this data are scarce. We developed a Python toolbox that specializes in processing high-resolution brain imaging data. It has been designed with two key purposes in mind:

i. to provide the neuroimaging community with user-friendly access to cutting-edge high-resolution image processing tools
ii. to create a flexible framework that can be extended by other researchers, along with thorough instructions on how to contribute

Comparison to other tools

Most major neuroimaging packages are optimized for data with a maximum spatial resolution of 1 mm isotropic. Only recently some extensions and new tools for processing of high-resolution data have begun to emerge, which will be discussed in the following.[20]

19 http://nighres.readthedocs.io/en/latest/auto_examples/index.html
20 cf. https://layerfmri.com/2018/01/04/layer-fmri-software-in-the-field/

Table 1. Computation times for usage example.

Processing step	Duration
Skull stripping	1 min 8 sec (8 sec)
MGDM tissue classification	6 min 58 sec (30 sec)
CRUISE surface reconstruction	1 min 57 sec (3 sec)
Equivolumetric layering	1 min 23 sec (6 sec)

Shown are average durations over 10 repetitions (with standard deviations in brackets), determined on a standard laptop. See main text for details.

Freesurfer

Freesurfer is a popular open-source package for analyzing cortical surface data [40, 41]. It is robust, well-documented and applicable across platforms. By default, Freesurfer resamples the input data to a spatial resolution of 1 mm isotropic, obliterating the advantages of higher resolution data. The latest Freesurfer release includes an option for processing at submillimiter resolution [42]. However, this option is still under development and tested only for a resolution of 0.75 mm.[21] A Matlab routine for laminar analysis of high-resolution MRI data using Freesurfer has been proposed as well.[22] Here, intracortical surfaces are evolved as triangular meshes starting from the gray-white matter boundary in an equidistant fashion. This approach can cause errors and mesh irregularities, especially closer to the pial surface, and does not take into account the known dependency of layer thickness on local curvature.

More generally, Freesurfer's robustness and ease-of-use come at the cost of strict requirements for data organization (e.g. imposed directory structure, native file format) and limited flexibility in the adaptation of individual processing steps for new applications. While it provides excellent pipelines for standard processing of T1- or T2-weighted whole brain scans, it is not optimized for processing non-standard data such as quantitative T1 maps or images with partial brain coverage. At the same time, replacing individual processing steps with customized algorithms, combining Freesurfer with other tools, or applying manual corrections can be challenging even for experienced users. Therefore, while Freesurfer will likely play an important role as ultra-high field imaging becomes more abundant, it currently lacks the flexibility required for the active and collaborative development of new techniques in this emerging field.

BrainVoyager

Another software that has recently extended its functionality to the specific demands of high-resolution image processing is BrainVoyager [43]. A new pipeline, comprehensively described in a recent publication [44], enables laminar and columnar analyses of 9.4T data. Intracortical surfaces are modeled following the equivolumetric approach through the evolution of regular grids for small regions of interest. Unfortunately, BrainVoyager is a commercial software with closed source code. Besides the financial aspect of buying a license, this also entails that details of the applied algorithms are not transparent and the software cannot be adapted by users.

LAYNII

LAYNII is a set of highly optimized C++ tools for laminar analysis of high-resolution fMRI data with partial brain coverage [12].[23] Equivolumetric layering is available for slices without 3D curvature. The implementation in C++ enables fast processing, but has the disadvantage that fewer researchers can adapt or contribute code, as compared to high-level languages like Python. LAYNII also lacks documentation, making it hard for new users to adopt it.

LAYNII is a good example of an advanced toolbox that serves a specific purpose and could benefit from being combined with a more comprehensive and well-documented software framework for high-resolution image processing. It will be crucial in the future to synchronize Nighres with more specialized projects such as LAYNII, and make their integration as easy as possible.

CBS Tools

Nighres evolved out of CBS Tools, a suite of Java tools providing dedicated open source methods for high-resolution and quantitative image processing [19]. This includes specialized techniques such as equivolumetric layering [35] or multi-modal surface registration [36], as well as versions of more common applications, like tissue classification, that have been optimized for high-resolution data and quantitative contrasts. While many standard processing algorithms in neuroimaging grow at a log-linear ($O(N \log N)$) or even quadratic ($O(N^2)$) rate with data size, CBS Tools' algorithms approach linear rates ($O(N)$) or use non-iterative solutions [for details see 19]. CBS Tools can thus routinely operate on data at resolutions of up to 0.5 mm isotropic.

As described in the introduction, CBS Tools' complex design and heavy dependencies make the installation and handling challenging and impede contributions from other researchers. In a previous project report we have presented simple Python wrappers for selected CBS Tools functions [45]. Here we described a comprehensive software framework that has evolved out of these initial attempts. With Nighres we present a flexible and user-friendly implementation of CBS Tools' functionality, which eliminates the dependency on MIPAV and JIST. This approach provides a significant improvement in usability while preserving the excellent performance of CBS Tools. Another major advance of Nighres compared to CBS Tools is its extensive online documentation. Besides explaining every function's in- and outputs, it provides carefully documented usage examples with step-by-step instructions of how the different tools can be combined to create complete processing pipelines. The implementation in Python along with a detailed developer's guide facilitate adaptation and extension of the existing tools by other researchers.

We gave an example of Nighres' performance in the previous section (see Table 1). To put this example into perspective, consider Freesurfer's `recon-all` command, probably the most common approach for whole brain tissue classification and cortical surface reconstruction. This command processes a whole brain image at 1 mm isotropic resolution within a few hours. In comparison, the Nighres pipeline presented above achieves tissue classification and segmentation **plus** cortical layering at 0.7 mm isotropic resolution (roughly corresponding to a three-fold increase in data size as compared to 1 mm isotropic) in less than 15 minutes.

Future directions

The current implementation of Nighres contains a set of cutting-edge methods, but rapid methodological advances are to be expected in the dynamic field of high-resolution neuroimaging. We therefore designed Nighres as a transparent software platform through which newly developed methods can be made available to the community and improved collaboratively. New or existing tools can easily be added in a variety

21 https://surfer.nmr.mgh.harvard.edu/fswiki/SubmillimeterRecon
22 https://github.com/kendrickkay/cvncode/blob/master/cvnmakelayers.m
23 https://github.com/layerfMRI

of formats, depending on the specific requirements of the operation and the preferences of the developer. The extensive developer's guide aims to encourage contributions even from researchers without extensive experience in software development.

We intend to closely integrate our package with the existing community around neuroimaging tools in Python. To this end, we adopted standardized objects for internal data handling, which can easily be exchanged with other tools. An example is the seamless visualization of Nighres outputs using Nilearn's [26] plotting functions, as showcased in the usage example (Figure 1 and 2).

A major limitation of the current package is that it has been developed and tested for common Linux platforms only. The C++ code generated by JCC to interface with CBS Tools' Java classes makes the compilation platform dependent. We addressed this issue by providing an automated build script that recompiles the code upon installation. While this process has only been tested on Linux, the design makes a future adaptation to Mac OS X platforms straightforward. Support for Windows is not currently planned. However, the provided Dockerfile enables usage of Nighres in a container on any platform that supports Docker.

Many future extensions of the current package can be envisioned. Besides integrating more of the original CBS Tools functions, a main goal is to extend functionality with new tools coded directly in Python and potentially to replace the Java dependency altogether. To ensure efficient processing of the large data this might require increasing Python's performance for example using Numba.[24] Another goal is to provide integration with tools for parallel processing and job management on compute clusters.

Conclusion

We developed a user-friendly and well-documented Python package that makes cutting-edge high-resolution image processing tools available to the research community. The toolbox is easy to install and provides a comprehensive set of advanced techniques. While the current functionality is largely based on CBS Tools, we hope that the flexible framework encourages contribution of new tools, stimulates collaboration, and accelerates progress in the promising field of high-resolution neuroimaging.

Availability and requirements

- Project name: Nighres
- Project home page: https://github.com/nighres/nighres
- Operating system(s): Linux
- Programming language: Python, Java
- Other requirements: Java≥1.7, Python≥2.7, Numpy≥1.13, Nibabel≥2.1.0
- License: Apache License 2.0
- RRID: SCR_016287

24 https://numba.pydata.org/

Availability of supporting data

The data sets supporting the results of this article are available in the NITRC image repository [29] under https://www.nitrc.org/frs/?group_id=1205. Snapshots of the data and code are also available in the GigaScience GigaDB repository [46].

Declarations

List of abbreviations

- CRUISE - cortical reconstruction using implicit surface evolution
- MGDM - multiple object geometric deformable model
- MPM - quantitative multi-parameter mapping
- MP2RAGE - magnetization prepared two rapid acquisition gradient echoes
- MRI - magnetic resonance imaging
- NITRC - the neuroimaging informatics tools and resources clearinghouse
- QSM - quantitative susceptibility mapping
- T - Tesla

Ethical Approval

Not applicable.

Consent for publication

Not applicable.

Competing Interests

The authors declare that they have no competing interests.

Funding

JMH project was partially funded by a stipend from Google via the Google Summer of Code 2017 program, with INCF as mentoring organization.

Author's Contributions

JMH, CJS and PLB contributed equally to the conceptualization of the project and writing of the manuscript. JMH lead and CJS and PLB supported software development. All authors read and approved the final manuscript.

Acknowledgements

We would like to thank Gilles de Hollander, Nathaniel Kofalt and Rüdiger Meier for their contributions to Nighres, Daniel S. Margulies for his continuous support of this project, and Malin Sandström and the INCF for their coordination of the Google Summer of Code project.

References

1. van der Zwaag W, Schäfer A, Marques JP, Turner R, Trampel R. Recent applications of UHF-MRI in the study of human brain function and structure: a review: UHF MRI: Applications to Human Brain Function and Structure. NMR in Biomedicine 2016;29(9):1274–1288.

2. Lutti A, Dick F, Sereno MI, Weiskopf N. Using high-resolution quantitative mapping of R1 as an index of cortical myelination. NeuroImage 2014;93:176–188.
3. Sereno MI, Lutti A, Weiskopf N, Dick F. Mapping the human cortical surface by combining quantitative T1 with retinotopy. Cereb Cortex 2013;23(9):2261–2268.
4. Dick F, Tierney AT, Lutti A, Josephs O, Sereno MI, Weiskopf N. In vivo functional and myeloarchitectonic mapping of human primary auditory areas. J Neurosci 2012;32(46):16095–16105.
5. Huntenburg JM, Bazin PL, Goulas A, Tardif CL, Villringer A, Margulies DS. A Systematic Relationship Between Functional Connectivity and Intracortical Myelin in the Human Cerebral Cortex. Cerebral Cortex 2017;27(2):981–997.
6. Dinse J, Härtwich N, Waehnert MD, Tardif CL, Schäfer A, Geyer S, et al. A cytoarchitecture-driven myelin model reveals area-specific signatures in human primary and secondary areas using ultra-high resolution in-vivo brain MRI. NeuroImage 2015;114:71–87.
7. Waehnert MD, Dinse J, Schäfer A, Geyer S, Bazin PL, Turner R, et al. A subject-specific framework for in vivo myeloarchitectonic analysis using high resolution quantitative MRI. NeuroImage 2016;125:94–107.
8. Fracasso A, van Veluw SJ, Visser F, Luijten PR, Spliet W, Zwanenburg JJM, et al. Lines of Baillarger in vivo and ex vivo: Myelin contrast across lamina at 7T MRI and histology. Neuroimage 2016;133:163–175.
9. Whitaker KJ, Vértes PE, Romero-Garcia R, Váša F, Moutoussis M, Prabhu G, et al. Adolescence is associated with genomically patterned consolidation of the hubs of the human brain connectome. Proc Natl Acad Sci U S A 2016;113(32):9105–9110.
10. Marques JP, Khabipova D, Gruetter R. Studying cyto and myeloarchitecture of the human cortex at ultra-high field with quantitative imaging: R1, R2(*) and magnetic susceptibility. Neuroimage 2017;147:152–163.
11. Kok P, Bains L, vanMourik T, Norris D, deLange F. Selective Activation of the Deep Layers of the Human Primary Visual Cortex by Top-Down Feedback. Current Biology 2016;26(3):371–376.
12. Huber L, Handwerker DA, Jangraw DC, Chen G, Hall A, Stüber C, et al. High-Resolution CBV-fMRI Allows Mapping of Laminar Activity and Connectivity of Cortical Input and Output in Human M1. Neuron 2017;96(6):1253–1263.e7.
13. Keuken MC, Bazin PL, Crown L, Hootsmans J, Laufer A, Müller-Axt C, et al. Quantifying inter-individual anatomical variability in the subcortex using 7T structural MRI. NeuroImage 2014;94:40–46.
14. Steele CJ, Anwander A, Bazin PL, Trampel R, Schaefer A, Turner R, et al. Human Cerebellar Sub-millimeter Diffusion Imaging Reveals the Motor and Non-motor Topography of the Dentate Nucleus. Cerebral Cortex 2017;27(9):4537–4548.
15. Thürling M, Kahl F, Maderwald S, Stefanescu RM, Schlamann M, Boele HJ, et al. Cerebellar cortex and cerebellar nuclei are concomitantly activated during eyeblink conditioning: a 7T fMRI study in humans. J Neurosci 2015;35(3):1228–1239.
16. Forstmann BU, Keuken MC, Schafer A, Bazin PL, Alkemade A, Turner R. Multi-modal ultra-high resolution structural 7-Tesla MRI data repository. Sci Data 2014;1:140050.
17. Gorgolewski KJ, Mendes N, Wilfling D, Wladimirow E, Gauthier CJ, Bonnen T, et al. A high resolution 7-Tesla resting-state fMRI test-retest dataset with cognitive and physiological measures. Sci Data 2015;2:140054.
18. Tardif CL, Schäfer A, Trampel R, Villringer A, Turner R, Bazin PL. Open Science CBS Neuroimaging Repository: Sharing ultra-high-field MR images of the brain. Neuroimage 2016;124:1143–1148.
19. Bazin PL, Weiss M, Dinse J, Schäfer A, Trampel R, Turner R. A computational framework for ultra-high resolution cortical segmentation at 7Tesla. Neuroimage 2014;93 Pt 2:201–209.
20. Bazin PL, Plessis V, Fan AP, Villringer A, Gauthier CJ. Vessel segmentation from quantitative susceptibility maps for local oxygenation venography. In: 2016 IEEE 13th International Symposium on Biomedical Imaging (ISBI); 2016. p. 1135–1138. doi: 10.1109/ISBI.2016.7493466.
21. McAuliffe MJ, Lalonde FM, McGarry D, Gandler W, Csaky K, Trus BL. Medical Image Processing, Analysis and Visualization in clinical research. In: Proceedings 14th IEEE Symposium on Computer-Based Medical Systems. CBMS 2001; 2001. p. 381–386. doi: 10.1109/CBMS.2001.941749.
22. Lucas BC, Bogovic JA, Carass A, Bazin PL, Prince JL, Pham DL, et al. The Java Image Science Toolkit (JIST) for rapid prototyping and publishing of neuroimaging software. Neuroinformatics 2010;8(1):5–17.
23. Muller E, Bednar JA, Diesmann M, Gewaltig MO, Hines M, Davison AP. Python in neuroscience. Front Neuroinform 2015;9:11.
24. Brett M, Hanke M, Cipollini B, Côté MA, Markiewicz C, Gerhard S, et al. nibabel: 2.1.0 (Version 2.1.0). Zenodo 2016;doi:10.5281/zenodo.60808.
25. Gorgolewski KJ, Burns CD, Madison C, Clark D, Halchenko YO. Nipype : a flexible, lightweight and extensible neuroimaging data processing framework in Python. Front Neuroinform 2011;5.
26. Abraham A, Pedregosa F, Eickenberg M, Gervais P, Mueller A, Kossaifi J, et al. Machine learning for neuroimaging with scikit-learn. Front Neuroinform 2014;8:14.
27. van der Walt S, Colbert SC, Varoquaux G. The NumPy Array: A Structure for Efficient Numerical Computation. Computing in Science Engineering 2011;13(2):22–30.
28. Bazin J P L Kipping, Steele CJ, Margulies D, Turner R, Villringer A, Subject-specific cortical cerebellar mapping at 3T and 7T;. Poster presented at the 19th Annual Meeting of the Organization for Human Brain Mapping, June 16-20, 2013, Seattle, USA. (Abstract #1788).
29. Kennedy DN, Haselgrove C, Riehl J, Preuss N, Buccigrossi R. The NITRC image repository. NeuroImage 2016;124(Part B):1069–1073.
30. Marques JP, Kober T, Krueger G, van der Zwaag W, Van de Moortele PF, Gruetter R. MP2RAGE, a self bias-field corrected sequence for improved segmentation and T1-mapping at high field. Neuroimage 2010;49(2):1271–1281.
31. Bogovic J, Prince J, Bazin P. A multiple object geometric deformable model for image segmentation. Computer Vision and Image Understanding 2013;117(2):145–157.
32. Fan X, Bazin PL, Prince JL. A multi-compartment segmentation framework with homeomorphic level sets. In: 2008 IEEE Conference on Computer Vision and Pattern Recognition; 2008. p. 1–6.
33. Han X, Pham DL, Tosun D, Rettmann ME, Xu C, Prince JL. CRUISE: Cortical reconstruction using implicit surface evolution. NeuroImage 2004;23(3):997 – 1012.
34. Bok S. Der Einfluß der in den Furchen und Windungen auftretenden Krümmungen der Großhirnrinde auf die Rindenarchitektur. Arch Psychiatr Nervenkr Z Gesamte Neurol Psychiatr 1929;12:682–750.
35. Waehnert MD, Dinse J, Weiss M, Streicher MN, Waehnert P, Geyer S, et al. Anatomically motivated modeling of cortical laminae. NeuroImage 2014;93 Pt 2:210–20.
36. Tardif CL, Schäfer A, Waehnert M, Dinse J, Turner R, Bazin PL. Multi-contrast multi-scale surface registration for improved alignment of cortical areas. NeuroImage

2015;111:107–122.
37. Bazin PL, Pham DL. Topology correction of segmented medical images using a fast marching algorithm. Computer Methods and Programs in Biomedicine 2007;88(2):182 – 190.
38. Trampel R, Bazin PL, Pine K, Weiskopf N. In-vivo magnetic resonance imaging (MRI) of laminae in the human cortex. Neuroimage 2017;doi: 10.1016/j.neuroimage.2017.09.037.
39. Paus T. Imaging microstructure in the living human brain: A viewpoint. NeuroImage 2017;doi: 10.1016/j.neuroimage.2017.10.013.
40. Dale AM, Fischl B, Sereno MI. Cortical Surface-Based Analysis: I. Segmentation and Surface Reconstruction. Neuroimage 1999;9(2):179–194.
41. Fischl B, Sereno MI, Dale AM. Cortical Surface-Based Analysis: II: Inflation, Flattening, and a Surface-Based Coordinate System. Neuroimage 1999;9(2):195–207.
42. Zaretskaya N, Fischl B, Reuter M, Renvall V, Polimeni JR. Advantages of cortical surface reconstruction using submillimeter 7 T MEMPRAGE. Neuroimage 2017;165:11–26.
43. Goebel R. BrainVoyager–past, present, future. Neuroimage 2012 Aug;62(2):748–756.
44. Kemper VG, Martino FD, Emmerling TC, Yacoub E, Goebel R. High resolution data analysis strategies for mesoscale human functional MRI at 7 and 9.4T. NeuroImage 2018;164:48 – 58.
45. Huntenburg JM, Wagstyl K, Steele C, Funck T, Ai R, Bethlehem OF, et al. Laminar Python: Tools for cortical depth-resolved analysis of high-resolution brain imaging data in Python. Research Ideas and Outcomes 2017;3.
46. Huntenburg JM, Steele CJ, Bazin PL. Supporting data for "Nighres: Processing tools for high-resolution neuroimaging". GigaScience Database 2018;Http://dx.doi.org/10.5524/100469.
