# Supplementary material for: Nighres: processing tools for high-resolution neuroimaging
Source: Gigascience. 2018 Jul 4;7(7):giy082. doi: 10.1093/gigascience/giy082 (PMC6065481; doi:10.1093/gigascience/giy082)
Supplement: Response_to_Reviewer_Comments_Original_Submission.pdf [file giy082_response_to_reviewer_comments_original_submission.pdf]

Author's Response To Reviewer Comments

Close

We have now included the reference to the GigaDB entry in the section "Availability of supporting data".

Close
