# Supplementary material for: Nighres: processing tools for high-resolution neuroimaging
Source: Gigascience. 2018 Jul 4;7(7):giy082. doi: 10.1093/gigascience/giy082 (PMC6065481; doi:10.1093/gigascience/giy082)
Supplement: Reviewer_1_Report_(Original_Submission) -- Laurentius Huber — 1/8/2018 Reviewed [file giy082_reviewer_1_report_(original_submission).pdf]

Reviewer Report

Title: **Nighres: Processing tools for high-resolution neuroimaging**

Version: **Original Submission** **Date:** 1/8/2018

Reviewer name: **Laurentius Huber**

Reviewer Comments to Author:

The manuscript entitled "Nighres: Processing tools for high-resolution neuroimaging" describes a software suite that manages existing CBS-tools for applications in Python and is designed to allow future incorporations from other researchers.The biggest strength of the manuscript is that it will serve a pressing need of a growing research field. Namely an open, straightforward, robust, and adaptable analysis toolbox for high-resolution imaging data. Another advantage of the described software tool is that it is fast and efficient even without multi-treating.The weakest point of the manuscript is that is does doesn't really contain any new 'science' or any novel 'algorithms'. The described software toolbox is just a different form of managing previously published code in different environments. Because of all the used wrappers and multi-language integration tools, I am not sure how straightforward it will be for future developers to incorporate new tools without learning the specific environment. I downloaded and installed it myself and I can confirm it's user friendly applicability on Unix systems. I want to congratulate the authors for the speed of the tools. For Mac, however, the installation requires more advanced background knowledge and/or additional overhead with the Docker-workaround. I want to express my enthusiastic support for the publication of the manuscript. Though, I think the authors should consider addressing the following points before publication.1. The manuscript shows strong similarities with a previous publication (Huntenburg et al., 2017). In order to avoid the impression that this is a "duplicate publication", I feel it would be appropriate to cite the older publications and/or explicitly pointing out the novel parts of the manuscript at hand. 2. Fig. 1C depicts a different axial slice compared to Fig. 1A and 1B. I would advise the authors to use a consistent depiction scheme across all panels. 3. I am not entirely convinced that alternative software packages for high-resolution MRI/fMRI analysis are appropriately acknowledged.On page 5, line 1, the authors claim that alternative software packages that are also being specialized for applications at high resolutions (e.g. Freesurfer), would suffer from "rigid data organization". It is not clear to me what this means and how this results in a disadvantage compared Nighres. If the authors insist on claiming that Nighres would be less limited than alternative software packages, I would advise the authors to expand this discussion.→ E.g. I believe, Kendrick Kay's optimized scripts of using Freesurfer for high-res layer MRI might be easier applicable across platforms. (https://github.com/kendrickkay/cvncode/blob/master/cvnmakelayers.m)→ E.g. The Brain Voyager pipeline from Valentin Kemper claims to be better suited for simultaneous columnar and laminar analyses (Kemper et al., 2017). → E.g. I believe the C++ tools from myself might be more optimized fro real-live application in fMRI of limited coverage https://github.com/layerfMRI (Huber et al., 2017). Hence, I feel, it would be appropriate to be more specific when discussing advantages of Nighres, rather then simply dismissing alternative packages as 'limited'. 4. The abstract of the manuscript promises the reader 'advanced techniques for high-resolution neuroimaging'. However, the discussed example in the main text deals with low level applications of scull stripping and segmentation only. Which is not really specific to high resolution. I think the manuscript would benefit from a more advanced example, e.g. the layering tools that are used as the main selling point in the introduction. Maybe the manuscript would be even more compelling, if the authors could refer the reader to more advanced examples form their documentation site. E.g. the layering example form their website: http://nighres.readthedocs.io/en/latest/auto_examples/example_cortical_depth_estimation.html#volumetric-layering 5. Stylistic comments: → Page 4, line 26: "worked well ,we plot" → "worked well, we plot" → Page 5, line 46: "Many future extension of" → "many future extensions of"→ citation 13 is referring to a conference abstract. The corresponding paper is published now (Huber et al., 2017). → citation 29 is lacking the abstract number.→ citation 33 is lacking a doi or page numbers. I believe the doi is:

10.1016/j.neuroimage.2017.09.037Laurentius Huber, Laurentius.Huber@nih.gov,Visiting Research FellowNational Institute of Mental HealthBethesda, USAI am happy to clarify comments if they are not clear.References:Huber, L., Handwerker, D.A., Jangraw, D.C., Chen, G., Hall, A., Stüber, C., Gonzalez-Castillo, J., Ivanov, D., Marrett, S., Guidi, M., et al. (2017). High-resolution CBV-fMRI allows mapping of laminar activity and connectivity of cortical input and output in human M1. Neuron 96, 1-11.Huntenburg, J., Wagstyl, K., Steele, C., Funck, T., Bethlehem, R., Foubet, O., Larrat, B., Borrell, V., and Bazin, P.-L. (2017). Laminar Python: tools for cortical depth-resolved analysis of high-resolution brain imaging data in Python. Res. Ideas Outcomes 3, e12346.Kemper, V.G., De Martino, F., Emmerling, T., Yacoub, E., and Goebel, R. (2017). 9.4 Tesla imaging and high resolution data analysis strategies for mesoscale human functional MRI. Neuroimage in press, doi:10.1016/j.neuroimage.2017.03.058.

Level of Interest

Please indicate how interesting you found the manuscript: An article of importance in its field

Quality of Written English

Please indicate the quality of language in the manuscript: Acceptable

Declaration of Competing Interests

Please complete a declaration of competing interests, considering the following questions:

- Have you in the past five years received reimbursements, fees, funding, or salary from an organisation that may in any way gain or lose financially from the publication of this manuscript, either now or in the future?
- Do you hold any stocks or shares in an organisation that may in any way gain or lose financially from the publication of this manuscript, either now or in the future?
- Do you hold or are you currently applying for any patents relating to the content of the manuscript?
- Have you received reimbursements, fees, funding, or salary from an organization that holds or has applied for patents relating to the content of the manuscript?
- Do you have any other financial competing interests?
- Do you have any non-financial competing interests in relation to this paper?

If you can answer no to all of the above, write 'I declare that I have no competing interests' below. If your reply is yes to any, please give details below.

I declare that I have no competing interests.

I agree to the open peer review policy of the journal. I understand that my name will be included on my report to the authors and, if the manuscript is accepted for publication, my named report including any attachments I upload will be posted on the website along with the authors' responses. I agree for my report to be made available under an Open Access Creative Commons CC-BY license

(http://creativecommons.org/licenses/by/4.0/). I understand that any comments which I do not wish to be included in my named report can be included as confidential comments to the editors, which will not be published.

I agree to the open peer review policy of the journal

To further support our reviewers, we have joined with Publons, where you can gain additional credit to further highlight your hard work (see: https://publons.com/journal/530/gigascience). On publication of this paper, your review will be automatically added to Publons, you can then choose whether or not to claim your Publons credit. I understand this statement. Yes
