# Supplementary material for: Nighres: processing tools for high-resolution neuroimaging
Source: Gigascience. 2018 Jul 4;7(7):giy082. doi: 10.1093/gigascience/giy082 (PMC6065481; doi:10.1093/gigascience/giy082)
Supplement: Reviewer_2_Report_(Original_Submission) -- Christin Y. Sander, Ph.D. — 2/15/2018 Reviewed [file giy082_reviewer_2_report_(original_submission).pdf]

Reviewer Report

Title: **Nighres: Processing tools for high-resolution neuroimaging**

Version: **Original Submission** **Date:** 2/15/2018

Reviewer name: **Christin Y. Sander**

Reviewer Comments to Author:

The manuscript by Huntenburg et al. is a Technical Note that presents a new python package "Nighres" for processing high-resolution neuroimaging data. The presented software package is based on a previously implemented processing tool software (CBS High-Res Brain Processing Tools) that contains a Python-wrapped interface of the available existing functions and can thus easily interface with pure Python code or other Python packages. This new interface is likely going to make the software accessible to a broader set of neuroimaging users. The paper itself is well-written and structured very logically. It explains the implementation of the python package clearly and gives a nice example of an image processing pipeline to obtain tissue classification from an anatomical image. One main part that is missing from the manuscript is a summary of the description of the functionality of the software, performance metrics or comparison to existing neuroimage processing tools. Including this would help make the manuscript a more complete description of the software and provide readers with a useful overview. Main comments: 1) Including a section with an overview of the functionality and performance of the software package: Although the purpose of this paper is to take an existing package, not all readers will be familiar with the previously developed CBS Tools. One example on tissue classification is given, which is very helpful, but a section outlining/summarizing currently available functions, and the performance in the context of existing software, would make this paper a more complete description. 2) In the introduction, a major argument is made that this software is specifically developed to be able to handle very large datasets. It would be helpful to include a more direct discussion of the arguments why this specific package is able to handle large datasets better than / or is equivalent to other popular neuroimaging software. This is linked to the point above, where the performance or functionality could be compared (quantitatively and/or qualitatively) to other available neuroimaging software packages. Alternatively, it should be clearly stated why this software is providing a new functionality not existing in other toolboxes. Additional specific comments: 3) Page 4, col 1, ll 15-19A little more background / explanation of the first paragraph will be helpful as it is not intuitive why both a quantitative T1 map and a T1-weighted image automatically gives a brain mask. 4) Page 4, col 1, l 26placement of the comma5) Page 5, col 1, l 37: "The C++ code generated by .. (to) interfaces''Sentence structure is not complete, please check. Comment on references: Some references contain either website or doi links, but not all. In addition, some references refer to conference proceedings that are not all publicly available, or searchable. Please review the references accordingly, and provide a website link with accessed date if needed. Please make sure to check for consistency and follow the guidelines of this journal.

Level of Interest

Please indicate how interesting you found the manuscript: An article whose findings are important to those with closely related research interests

Quality of Written English

Please indicate the quality of language in the manuscript: Acceptable

Declaration of Competing Interests

Please complete a declaration of competing interests, considering the following questions:

- Have you in the past five years received reimbursements, fees, funding, or salary from an organisation that may in any way gain or lose financially from the publication of this manuscript, either now or in the future?
- Do you hold any stocks or shares in an organisation that may in any way gain or lose financially from the publication of this manuscript, either now or in the future?
- Do you hold or are you currently applying for any patents relating to the content of the manuscript?
- Have you received reimbursements, fees, funding, or salary from an organization that holds or has applied for patents relating to the content of the manuscript?
- Do you have any other financial competing interests?
- Do you have any non-financial competing interests in relation to this paper?

If you can answer no to all of the above, write 'I declare that I have no competing interests' below. If your reply is yes to any, please give details below.

I declare that I have no competing interests.

I agree to the open peer review policy of the journal. I understand that my name will be included on my report to the authors and, if the manuscript is accepted for publication, my named report including any attachments I upload will be posted on the website along with the authors' responses. I agree for my report to be made available under an Open Access Creative Commons CC-BY license (http://creativecommons.org/licenses/by/4.0/). I understand that any comments which I do not wish to be included in my named report can be included as confidential comments to the editors, which will not be published.

I agree to the open peer review policy of the journal

To further support our reviewers, we have joined with Publons, where you can gain additional credit to further highlight your hard work (see: https://publons.com/journal/530/gigascience). On publication of this paper, your review will be automatically added to Publons, you can then choose whether or not to claim your Publons credit. I understand this statement. Yes
